# Organoid‐Based Human Stomach Micro‐Physiological System to Recapitulate the Dynamic Mucosal Defense Mechanism

**DOI:** 10.1002/advs.202300164

**Published:** 2023-07-31

**Authors:** Hye‐Jin Jeong, Ji‐Hyeon Park, Joo H. Kang, Jonathan Sabaté del Río, Seong‐Ho Kong, Tae‐Eun Park

**Affiliations:** ^1^ Department of Biomedical Engineering Ulsan National Institute of Science and Technology Ulsan 44919 Republic of Korea; ^2^ Department of Surgery Seoul National University Hospital Seoul National University College of Medicine Seoul 03080 Republic of Korea; ^3^ Department of Surgery Gachon University Gil Medical Center Incheon 21565 Republic of Korea; ^4^ Center for Soft and Living Matter Institute for Basic Science (IBS) Ulsan 44919 Republic of Korea

**Keywords:** gastric mucosal defense mechanism, *Helicobacter pylori*, human gastric organoids, micro‐physiological system, telocytes, Trefoil factor 1

## Abstract

Several stomach diseases are attributed to the dysregulation of physiological function of gastric mucosal barrier by pathogens. Gastric organoids are a promising tool to develop treatment strategies for gastric infections. However, their functional features of in vivo gastric mucosal barrier and host–microbe interactions are limited due to the lack of physiological stimuli. Herein, a human stomach micro‐physiological system (hsMPS) with physiologically relevant gastric mucosal defense system is described based on the combination of organoid and MPS technology. A fluid flow enhanced epithelial‐mesenchymal interaction in the hsMPS enables functional maturation of gastric epithelial cells, which allows for the recreation of mesh‐like mucus layer containing high level of mucus protective peptides and well‐developed epithelial junctional complexes. Furthermore, gastroprotection mechanisms against *Helicobacter pylori (H. pylori)* are successfully demonstrated in this system. Therefore, hsMPS represents a new in vitro tool for research where gastric mucosal defense mechanism is pivotal for developing therapeutic strategies.

## Graphical abstract

Gastric organoids have emerged as a promising tool to study gastric infection; however, they possess disadvantages in recreating complex mucosal defense system. This study proposes that microphysiological system (MPS) technology promotes the maturation of gastric organoids with physiologically relevant features of mucosal barrier against *H. pylori* including TFF1‐mediated biological fence, which allows the development of effective therapeutic approach.

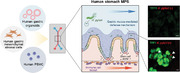

## 1 Introduction

The human gastric mucosal barrier is composed of epithelial, mucosal, and submucosal elements that play gastroprotective roles by blocking pathogens and harmful substances.^[^ ^1^ ^]^ The gastric epithelial barrier is maintained by continuous renewal of gastric stem cells within a stem cell niche where increased canonical wingless/integrated (Wnt) and Notch signaling activity and high contents of growth factors, such as fibroblast growth factor 2 (FGF2) and epidermal growth factor (EGF), are sustained.^[^ ^2^ ^]^ The surrounding mesenchymal stromal cells (gMSCs), including telocytes, influence the gastric epithelium function as a key regulator of the gastric stem cell proliferation and differentiation.^[^ ^2^, ^3^ ^]^ Gastric stem cells differentiate into progenitor cells, so‐called transit‐amplifying cells, that rapidly proliferate several times, and specialized to functional pit cells, which contribute to the overall function of the stomach, including secretion of mucus elements for gastric protection and controlling the stomach's pH environment.^[^ ^4^ ^]^ The gastric epithelium is covered by a mucus layer, which acts as a protective viscoelastic barrier composed of highly glycosylated mucin proteins forming a net‐like polymer, as well as protective peptides including trefoil factor peptides (TFF1 and TFF2).^[^ ^5^ ^]^ In the submucosal compartment, mucosal blood‐flow supplies the mucosa with nutrients and oxygen and removes metabolic waste to maintain the mucosal barrier and provide circulating immune cells for gastric immune responses against pathogens.

The homeostatic disruption of the gastric mucosal defense system against various foreign agents passing through the stomach leads to various kinds of stomach disease pathogenesis. For example, *Helicobacter pylori* infection is a leading cause of peptic ulcer disease, chronic gastritis, and stomach cancer, as it mediates a compromised gastric mucosal barrier, thereby affecting the integrity of the stomach.^[^ ^1^ ^]^ Therefore, understanding the gastric mucosal defense mechanisms against pathogens, and how pathogens evade or exploit the physiological defense system of the host, is important for development of effective treatment strategies against gastric diseases.

3D gastric organoids derived from stomach tissue are a promising tool for treatment development against gastric infections and disorders^[^ ^6^ ^]^ since they contain self‐renewing gastric stem cells that differentiate into multiple gastric‐specific epithelial cell types embedded in an extracellular matrix (ECM) hydrogel.^[^ ^7^ ^]^ 3D gastric organoids are more physiologically relevant than stomach cell lines since they mimic physiological epithelial regeneration process^[^ ^6a^ ^]^ and some aspects of *H. pylori* pathogenesis.^[^ ^8^ ^]^ However, the current gastric organoid systems have clear disadvantages in recreating the complex gastric mucosal defense system. In human gastric tissue, various niche factors in gastric glands help to maintain orchestrated stem cell proliferation and differentiation to provide functional epithelial cells constituting the gastric barrier.^[^ ^9^ ^]^ In gastric organoid culture, however, proliferation and differentiation of the gastric stem cells are simply regulated by the addition or withdrawal of Wnt signaling molecules (Wnt3a and R‐spondin1) in the culture media, which results in unbalanced generation of gastric epithelial cells.^[^ ^6^ ^]^ In addition, organoids are composed only of epithelial cells and do not contain immune cells. Cellular interaction with immune cells has been modeled by addition of specific immune cell populations such as neutrophils.^[^ ^10^ ^]^ The enclosed lumen structure of gastric organoids also hampers access to the apical side of the gastric epithelium and excretion of metabolic wastes from pathogens and epithelial cells, which is an inaccurate representation of the cell responses to pathogens.^[^ ^2^ ^]^ Previous studies generated a planar culture of the gastric epithelium in Transwell systems^[^ ^6^, ^9^ ^]^ to efficiently perform the inoculation of *H. pylori* to the apical side of the epithelium; however, this approach did not fully reflect gastric epithelial physiology, which requires continuous renewal of the epithelium by gastric stem cells and generation of differentiation progeny. The immature characteristics of gastric organoids, and technical difficulties associated with the model system, necessitates the need to develop technologies that promote the maturation of gastric organoids with physiologically relevant features of the gastric mucosal barrier and host–microbe interactions.

Recently, considerable efforts have been made to combine organoid technology and micro‐physiological systems (MPSs) to develop a more powerful in vitro platform with a synergistic combination of their features.^[^ ^11^ ^]^ MPS is a microfabricated 3D cellular construct designed to recapitulate the functions of organs through engineered microenvironment control.^[^ ^11^ ^]^ Implantation of brain,^[^ ^12^ ^]^ intestinal,^[^ ^13^ ^]^ ocular,^[^ ^14^ ^]^ and kidney organoids^[^ ^15^ ^]^ in MPS devices has led to greater functionality of organoids owing to controlled biochemical and biophysical microenvironmental cues and dynamic cell–cell interplay.^[^ ^16^ ^]^ An MPS system providing peristaltic flow through enclosed gastric organoids has been previously developed for efficient luminal delivery of nutrients or pharmacological agents.^[^ ^16^ ^]^ However, the implanted gastric organoid showed no physiological improvement, thereby demonstrating the biological and engineering‐design constraints of the past gastric MPS.

Herein, we describe a human stomach MPS (hsMPS), based on the combination of MPS technology and organoids, with a greatly enhanced gastric mucosal barrier. The hsMPS with epithelial cells, derived from human antral organoids (hAOs), and primary gMSCs, extracted from stomach tissue, are cultivated under controlled flow recapitulates gastric epithelial homeostasis for maintenance of the functional mucosal barrier. The resulting hsMPS exhibits a physiologically relevant mucosal defense system, including a mesh‐like mucus layer, which continuously covers the gastric mucosa and contains TFF peptides, and enhanced gastric epithelial junctional complexes. Furthermore, an *H. pylori* infection model was established in the hsMPS to monitor the dynamic mucosal defense mechanism against *H. pylori* infections, focusing on the critical role of TFF1.

## 2 Results and Discussion

### 2.1 Reconstitution of the Orchestrated Gastric Epithelial Homeostasis in an MPS System

**Figure 1:**
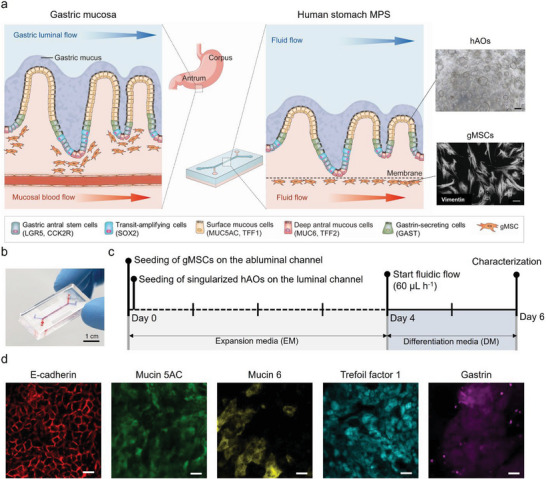
Reconstitution of the human stomach in an MPS device. a) Schematic illustration of the human gastric mucosa (left) and the human stomach MPS (hsMPS) with a singularized human gastric antral organoid (hAOs) cultured on the apical luminal channel interfaced with primary gastric stromal cells (gMSCs) on the basal abluminal channel (center). At the top right, a bright field image of hAOs (bar, 250 µm) used to generate gastric epithelium, and at the bottom right, an immunofluorescence micrograph of the gMSCs labeled with Vimentin (bar, 100 µm) are shown. b) Photograph of the hsMPS (bar, 1 cm). c) Timeline for the reconstitution of the human stomach in an MPS device. d) Immunofluorescence micrographs of the gastric epithelium in the hsMPS at 6 days labeled with E‐cadherin, mucin 5AC, mucin 6, trefoil factor 1, and gastrin (bar, 20 µm).

In gastric tissue, a stem cell divides asymmetrically, and give rise to a daughter stem cell and a highly proliferative gastric progenitor cell, that possess capacity to rapidly amplify the pool of differentiated.^[^ ^4^ ^]^ As a balance between self‐renewal and differentiation of stem cell is key to the formation of the gastric mucosal barrier, microenvironmental cues for gastric epithelial homeostasis were explored to generate the functionally enhanced gastric organoid‐based model (**Figure** **1**a). The gMSCs, including telocytes, have been suggested to play a critical role in maintaining activity of stem cell and progenitor cells as an important gastric stem cell niche.^[^ ^3^, ^9^, ^17^ ^]^ We hypothesized that co‐cultivation of gastric epithelial cells with gMSCs could recreate the microenvironment that orchestrates the gastric epithelial homeostasis by maintaining the proliferative activity of gastric progenitor cells in differentiation media (DM) conditions where differentiation progeny can be actively generated. Although it has been documented that gMSCs sustain the proliferative activity of gastric epithelial cells in tissue, maintaining their capacity in vitro is critical in achieving our goal. We assumed that mimicking mucosal blood flow delivery of nutrients and elimination of waste would improve the functional maintenance of gMSC,^[^ ^13^, ^18^ ^]^ as well as gastric epithelium,^[^ ^19^ ^]^ and facilitate epithelial–mesenchymal interactions.

To explore these possibilities, a compartmentalized polydimethylsiloxane (PDMS) device with luminal and abluminal microchannels separated by a porous polyethylene terephthalate (PET) membrane, which permits dynamic cell‐cell crosstalk and fluid flow control, was used in this study (Figure 1b). The human stomach was reconstituted via an MPS device by culturing singularized hAOs on the luminal (upper) microchannel interfaced with autologous primary gMSCs on the abluminal (lower) channel. The hAOs were specifically used to model the gastric antral mucosa, where the initial *H. pylori* colonization process occurs, to study host–bacterial interactions at an early stage. We first plated gMSCs in a lower microchannel and the device was flipped immediately to allow gMSCs attachment to the bottom of the PET membrane. Two hours after gMSCs seeding, gastric epithelial cells prepared by singularization of the hAOs were seeded on the apical channel and cultured for 4 days under static conditions in expansion media (EM). On day 5, Wnt protein‐deprived DM was introduced to the luminal and abluminal channels at a continuous flow of 60 µL h^−1^ (Figure 1c).^[^ ^13^, ^18^ ^]^ Confocal fluorescence microscopic imaging, conducted on day 6, revealed a distinct epithelial monolayer on the PET membrane in the luminal microchannel with well‐developed junctional complexes, which contained E‐cadherin along the lateral borders (Figure 1d). Gastric surface mucous cells secreting MUC5AC and TFF1, and deep mucous gland cells secreting MUC6, were also successfully identified in hsMPS (Figure 1d). Furthermore, gastrin‐producing G cells, found only in the gastric antral region, were detected using immunostaining for gastrin (Figure 1d), proving that multiple types of functional gastric epithelial cells were successfully generated in the hsMPS system.

### 2.2 Gastric Epithelial Cells Sustain Gastric Stem Cell Activity in the hsMPS

**Figure 2:**
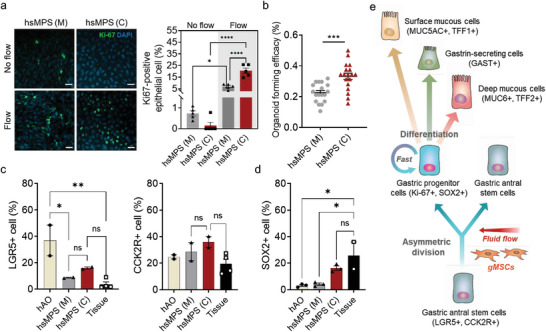
Sustainable gastric stem cell activity in the hsMPS. a) Immunofluorescence images of Ki‐67 in gastric epithelial cells (bar, 20 µm) (left) in the hsMPS in monocultures (M) and co‐cultures (C) under static (top) and flow conditions (bottom). Percentage of Ki‐67‐positive epithelial cells analyzed using ImageJ under static and flow conditions (right). b) Organoid forming efficacy assay in the hsMPS in monocultures (M) and co‐cultures (C) under flow conditions. c,d) Flow cytometry analysis of LGR5‐ (left), CCK2R‐ (right) positive gastric epithelial cells representing gastric stem cells; and SOX2‐positive gastric progenitor cells in the hsMPS in monocultures (M) and co‐cultures (C) under flow conditions compared to hAOs. e) Schematic diagram illustrating the orchestrated gastric epithelial proliferation and differentiation in the hsMPS by mesenchymal niche factors derived from gMSCs under fluid flow. The results are presented as the mean ± SEM. For statistical analysis, a one‐way ANOVA and Tukey's test was performed (*p < 0.05; **p < 0.01; ***p < 0.001; ****p < 0.0001). ns: non‐significant.

We determined whether co‐culturing of gMSCs in the presence of fluid flow in an MPS device would facilitate proliferative capacity of gastric epithelium in an MPS device, which is essential for constant supply of progenitor cells to maintain the gastric barrier. Confocal microscopy of Ki‐67 was conducted to examine the mitotic activity of epithelium in flow versus static conditions and in hsMPS (C) versus hsMPS (M) (**Figure** **2**a). In both mono‐ and co‐culture conditions, exposure to fluid flow exhibited significantly higher levels of Ki‐67 in gastric epithelial cells, indicating that fluid flow stimulated mitotic activity of gastric epithelial cells (Figure 2a). Earlier research has shown that continuous flow alone can enhance the proliferative ability of intestinal epithelial cells, including the Caco‐2 cell line,^[^ ^20^ ^]^ and primary small^[^ ^21^ ^]^ or large intestinal epithelial cells^[^ ^22^ ^]^ in microfluidic devices. Our study findings are consistent with these results. It has been proposed that basal flow can eliminate Wnt Antagonists such as DKK‐1 that are secreted to the basal side of gut epithelial cells. These findings suggest that the effect of fluid flow in increasing cell proliferation by up to seven times in hsMPS (M) may be attributed to the proper elimination of metabolic waste that could otherwise impede the maintenance of gastric epithelial cell proliferation, as well as the continuous provision of fresh media.

Remarkably, a large increase in the percentage of Ki‐67‐positive epithelial cells was observed in co‐culture versus mono‐culture conditions (20 ± 5.6% versus 6.9 ± 1.9%) only when exposed to fluid flow (Figure 2a). To confirm the facilitated mitotic activity of gastric epithelial cells under these conditions, epithelial cells were harvested from hsMPSs at day 6 and reseeded in a Matrigel droplet. The cells differentiated in an MPS device co‐cultured with gMSCs showed significantly higher organoid forming efficacy compared with monocultures in the presence of fluid flow (Figure 2b).

Next, we determined whether gMSCs and fluid flow would increase the number of gastric epithelial stem cells in DM. We conducted a flow cytometry analysis to quantify the composition of LGR+ and CCK2R+ cells in the hsMPSs compared to hAOs and gastric antrum tissue (Figure 2c; Figure S1, Supporting Information). LGR5‐ and CCK2R‐positive cells are long‐lived stem cells that reside in the deep base of the antral glands responsible for maintaining gastric epithelial homeostasis.^[^ ^5^ ^]^ Interestingly, when exposed to fluid flow, the percentage of LGR5+ or CCK2R+ cells in the hsMPS (C) did not show any significant changes compared to the hsMPS (M) (Figure 2c). This suggests that the presence of gMSCs and the cultivation of epithelial cells under fluid flow conditions do not alter the ratio of stem cells in DM, while it enhances the proliferative activity of epithelial cells (Figure 2a). Based on this result, we hypothesized that these conditions may facilitate the asymmetric division of gastric stem cells, leading to the generation of a daughter stem cell and a highly proliferative gastric progenitor cell, representing the initial step in stem cell differentiation. To further investigate this, we quantified the composition of cells expressing SOX2, a key marker for gastric progenitor cells,^[^ ^23^ ^]^ and found a fourfold increase in the percentage of gastric progenitor cells in the hsMPS (C) compared to the hsMPS (M), which indicates a substantially more abundant cellular source for differentiated progeny (Figure 2d). Importantly, the ratio of LGR+, CCK2R+, and SOX2+ cells in the gastric epithelial cells of the hsMPS (C) in DM closely resembled that of human gastric tissue, whereas conventional hAO cultures exhibited an overabundance of LGR5+ cells or a lower number of SOX2+ cells compared to the in vivo condition (Figure 2c). In the static conditions of hsMPS (C), the percentages of LGR5+ and SOX2+ were notably reduced compared to the flow conditions (Figure S1, Supporting Information), highlighting the significance of fluid flow. This finding suggests that the mesenchymal niche stimulated by fluid flow enables a balanced self‐renewal and differentiation of gastric stem cells in DM, resembling the gastric tissue environment (Figure 2e).

### 2.3 Fluid Flow‐Enhanced Niche Function of gMSC

**Figure 3:**
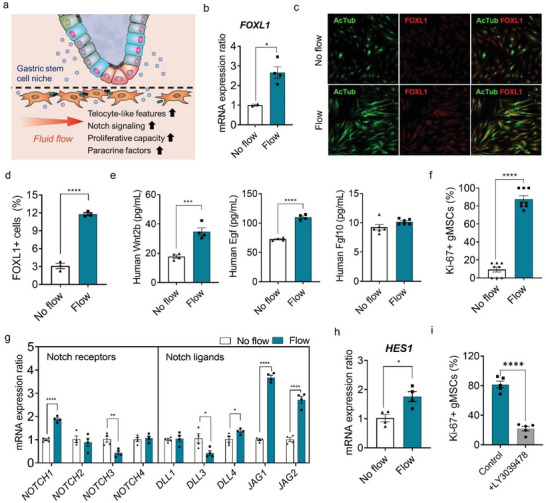
Fluid flow‐enhanced cellular niche function of gMSC and telocyte‐like morphogenesis. a) Schematic illustration of fluid flow‐enhanced niche function of gMSC. b) The mRNA expression ratios of genes encoding *FOXL1* of gMSCs in flow versus static conditions. c) Immunofluorescence micrographs of the gMSCs in the hsMPS labeled with acetylated tubulin marker (AcTub, green) and telocyte‐specific marker (FOXL1, red) (bar, 100 µm) (left). d) Quantification of Foxl1‐positive gMSCs using a flow cytometer under static and flow conditions. e) Quantification of human Wnt2b, Egf, and Fgf10 levels using ELISA in the media collected from hsMPS (C) under static and flow conditions. f) Percentage of Ki‐67‐positive gMSCs analyzed using ImageJ under static and flow conditions. g) mRNA expression ratio of Notch receptors (*NOTCH1*, *NOTCH2*, *NOTCH3*, and *NOTCH4*), Notch ligands (*DLL1*, *DLL3*, *DLL4*, *JAG1*, and *JAG2*). h) The mRNA expression ratio of transcription factor *HES1* initiated by the Notch signaling pathway in gMSCs under static and flow conditions. i) Percentage of Ki‐67‐positive gMSCs analyzed using ImageJ when treated with a Notch inhibitor, LY3039478 (100 nm). The results are presented as the mean ± SEM. For statistical analysis, a Student's *t*‐test was performed (*p < 0.05; **p < 0.01; ***p < 0.001; ****p < 0.0001).

Given that the in vivo relevant homeostasis of gastric stem cells is observed specifically when they are co‐cultivated with gMSCs in the presence of fluid flow, we further explored whether fluid flow affects the mesenchymal niche functions of gMSCs. When monitoring morphological changes of gMSCs in the hsMPSs under fluid flow, we found that gMSCs possess exceptionally long moniliform cytoplasmic processes forming mesh‐like networks (Figure S2, Supporting Information). Remarkably, such features are similarly observed in telocytes, which constitute a widespread interstitial meshwork in the lamina propria of the gastrointestinal tract. Telocytes are a major Wnt source in the stomach, supporting local stem cell niche maintenance and differentiation in development and tissue regeneration.^[^ ^3^, ^17^ ^]^ We hypothesized that fluid flow induced telocyte‐like features in gMSCs, thereby upregulating the expression of paracrine factors for maintaining proliferative capacity of gastric epithelial cells in DM conditions (**Figure** **3**a). To explore this possibility, we examined *FOXL1* expression in gMSCs, a key telocyte marker, using qRT‐PCR and confocal immunofluorescence microscopy. Our findings demonstrate that exposure to fluid flow substantially increased *FOXL1* mRNA and protein expression in gMSCs in the MPS device (Figure 3b,c). Furthermore, a flow cytometry analysis revealed a significant increase in the population of Foxl1‐positive gMSCs under flow conditions compared to static conditions (11.63% vs 3.19%) (Figure 3d; Figure S2, Supporting Information).

We further monitored differential secretion of paracrine factors, such as Wnt ligand (Wnt2b), epithermal growth factor (Egf), and fibroblast growth factor 10 (Fgf10),^[^ ^5^ ^]^ which regulate the proliferation of gastric stem cells and progenitor cells within the stem cell niche. The concentration of Wnt2b and Egf in the media collected from hsMPS (C) for 24 h was significantly higher under flow conditions compared to static conditions, while the level of Fgf10 secretion remained unchanged (Figure 3e). Importantly, the total volume of effluent collected from hsMPS (C) under flow conditions was 30 times higher than the collected media under static conditions, indicating that fluid flow activated gMSCs to continuously secrete high levels of Wnt2b, Egf, and Fgf10. It is worth noting that there may be other niche factors, including growth factors, hormones, and extracellular vesicles, that could also play a role in promoting epithelial proliferation, and thus cannot be ruled out.

Considering that gMSCs located in the lamina propria are not exposed to laminar fluid flow under normal conditions in gastric tissue, we hypothesize that fluid flow serves as a mechanical cue for gMSCs guiding tissue regeneration by obtain telocyte‐like features. Considering that molecular mechanism of the mesenchymal niche factor surrounding the gastric glands is poorly understood, further efforts are needed to elucidate interactions between gastric stem cells and gMSCs (or telocytes) for better recapitulation of gastric epithelium homeostasis in the hsMPS.

MSCs such as telocytes possess stem cell attributes that include a proliferative capacity, however, maintaining their characteristics in vitro is challenging.^[^ ^24^ ^]^ Through the Ki‐67 assessment, we confirmed that approximately 90% of gMSCs maintained mitotic activity when exposed to fluid flow in an MPS device, while those under static conditions lost their proliferative capacity (Figure 3f; Figure S2, Supporting Information). This resulted in an approximately 2.5 times higher number of gMSCs in the device under fluid flow (Figure S2, Supporting Information). Additionally, confocal immunofluorescence microscopic analysis of human gastric antral tissue verified the expression of Ki‐67 protein in Foxl1+ gMSCs, which is similar to the gMSCs that displayed proliferative potential in the microfluidic device (Figure S2, Supporting Information).

Notch activation in gMSCs, which is an important regulator of MSC maintenance,^[^ ^24^ ^]^ was further assessed when cultured within the device. Notably, we observed flow‐enhanced Notch signaling factors by comparing the gene expression levels of Notch ligands and receptors in gMSCs in flow versus static conditions. Statistically significant increases in mRNA expression were observed for genes encoding activating Notch ligands *JAG1*, *JAG2*, and *DLL4*, and Notch receptor *NOTCH1*, whereas the expression of the inhibitory Notch ligand *DLL3* was decreased (Figure 3g). Accordingly, *HES1*, a main downstream Notch target gene that inhibits cell senescence and maintains stemness of gMSCs,^[^ ^24^ ^]^ was significantly upregulated (Figure 3h). Furthermore, treating gMSCs with LY3039478, a Notch inhibitor, in the hsMPS(C) under flow conditions resulted in a 75% reduction in Ki‐67+ gMSCs, indicating the critical role of activated Notch signaling in maintaining gMSCs in fluid flow (Figure 3i; Figure S2, Supporting Information). These data reveal how fluid flow enhances the function of mesenchymal components in facilitating gastric epithelial homeostasis in vitro, which highlights the advantage of using the microfluidic system.

### 2.4 Formation of In Vivo‐Relevant Mucosal Barrier with gMSC Co‐Culture under Flow Conditions

**Figure 4:**
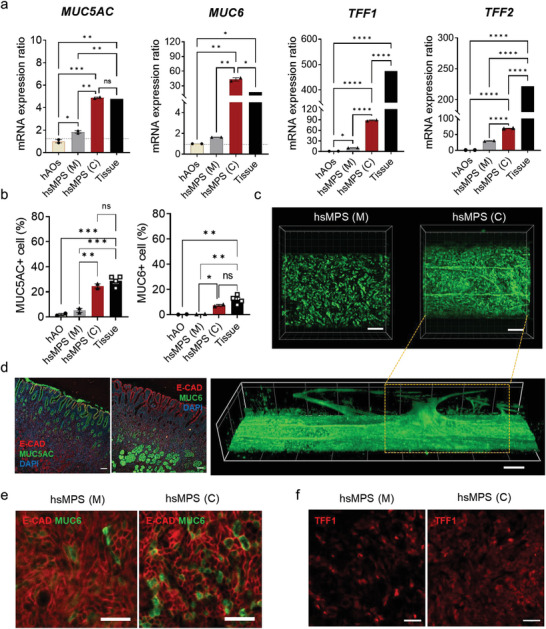
Recapitulation of an in vivo‐relevant gastric mucous barrier in the hsMPS. a) mRNA expression ratios of genes encoding mucin‐associated markers MUC5AC, MUC6, TFF1, and TFF2 in the gastric epithelial cells in monocultures (M) and co‐cultures (C) conditions in the hsMPS in the presence of fluid flow compared with hAOs. b) Flow cytometry analysis of MUC5AC+ and MUC6+ cells in gastric epithelial cells in the hsMPS in monocultures (M) and co‐cultures (C) under flow conditions compared to hAOs. c) Immunofluorescence micrograph of gastric epithelium labeled with mucin 5AC (green) under monoculture (top, left) and co‐culture (top, right) conditions (bar, 200 µm). 3D z‐stack image of mucin 5AC‐labeled mucus layers in the hsMPS (C) (bottom). d) Immunofluorescence micrograph of human gastric antral tissue labeled with E‐cadherin (E‐CAD, red), mucin 5AC (left, green), and mucin 6 (right, green) (bar, 100 µm). e) Immunofluorescence micrographs of epithelial cells stained with mucin 6 (green) and E‐cadherin (red) in monoculture (top) and co‐culture (bottom) (bar, 50 µm). f) Immunofluorescence images of gastric epithelial cells under monoculture and co‐culture conditions labeled with TFF1 (red) (bar, 50 µm). The results are presented as the mean ± SEM. For statistical analysis, a one‐way ANOVA and Tukey's test was performed (*p < 0.05; **p < 0.01; ***p < 0.001; ****p < 0.0001). ns: non‐significant.

We initially explored microenvironmental cues to achieve orchestrated gastric epithelial homeostasis in the hsMPS that are a key factor in recapitulating the formation and maintenance of the gastric mucosal barrier. After demonstrating the contribution of fluid flow in the epithelial‐mesenchymal interaction in the hsMPS, we focused on the role of gMSCs in the recreation of the gastric mucosal defense system in an MPS device under flow conditions. We first observed the mRNA expression of genes encoding major gastric mucus components (**Figure** **4**a). The highly glycosylated‐gel‐forming mucins of the stomach, including MUC5AC mucin, which expresses in the superficial gastric epithelium, and MUC6 mucin found in gastric glands physically protect the underlying gastric epithelium against noxious agents.^[^ ^25^ ^]^ TFF peptides (TFF1 and TFF2) co‐expressed with mucin proteins function as a chemical barrier in the gastric mucus layer by interacting with pathogens bound to the mucins.^[^ ^5^ ^]^ The TFF1 offers a binding moiety for *H. pylori* at the gastric antrum mucus layer and protects the gastric epithelium during *H. pylori* infection.^[^ ^26^ ^]^ Meanwhile, TFF2 plays a critical role in maintaining gastric mucosal integrity and exerts antibiotic function against pathogens entering the stomach.^[^ ^27^ ^]^ High similarity to the in vivo gastric mucous barrier in the hsMPSs was observed when mucin‐related gene expression of gastric epithelial cells was compared with that of hAOs (Figure 4a). We found statistically significant increases in mRNA expression of genes encoding MUC5AC, MUC6, TFF1, and TFF2 in the hsMPS compared with hAOs (Figure 4a). Furthermore, the mRNA levels of those genes were significantly upregulated and better matched with the in vivo gastric tissue in gastric epithelial cells when co‐cultured with gMSCs compared with hsMPS (M), as expected (Figure 4a). Interestingly, the mRNA expression of genes encoding TFF1 and TFF2 was increased in hsMPS (C), although their expression was still lower compared with in vivo tissues (Figure 4).

Remarkably, the ratios of MUC5AC‐positive (TFF1+) pit cells and MUC6‐positive (TFF2+) gland cells in the hsMPS (C) on fluidic culture, were close those in human gastric antral tissues, when analyzed using a flow cytometry (Figure 4b; Figure S1, Supporting Information). In agreement with the qRT‐PCR data, the percentage of MUC5AC‐positive cells was significantly higher in hsMPS (C) (24.7%) compared to hAOs (2.04%) or hsMPS (M) (5.24%) (Figure 4b; Figure S1, Supporting Information). Similarly, a significantly higher ratio of MUC6‐positive cells was observed in hsMPS (C) (7.35%) compared to hAOs (0.25%) or hsMPS (M) (0.51%). Importantly, in the hsMPS (C) under static conditions, the percentage of MUC5AC+ and MUC6+ cells were significantly decreased, further highlighting the synergistic effect of co‐cultivating with gMSCs and fluid culture in the device (Figure S1, Supporting Information). The ability to maintain the proliferation of gastric progenitor cells in these conditions resulted in a significant increase in the pool of fully differentiated cells in the hsMPS (C).

Confocal microscopic analysis confirmed that upregulated MUC5AC successfully formed the mesh‐like mucin network on superficial gastric epithelium in hsMPS (C) (Figure 4c) in an MPS device,^[^ ^28^ ^]^ similar to gastric tissue (Figure 4d). The expression of MUC5AC was also confirmed in hsMPS (M), however, formation of the mucin network was not observed (Figure 4c; Figure S3, Supporting Information). These results show that gMSCs enhanced the dynamic balance of production, secretion, and polymerization of mucin in the MPS under fluid flow. A higher production of MUC6 mucin protein was also identified using immunofluorescence (Figure 4e), which revealed that epithelial cells were also efficiently differentiated in deep mucous gland cells. In agreement with qRT‐PCR data, higher expression of TFF1 was also confirmed in the hsMPS (C) (Figure 4f). Considering that the current in vitro gastric models, including hAOs and human stomach cell lines, express extremely low TFF peptide levels, which necessitates transfection‐mediated TFF overexpression.^[^ ^29^ ^]^ Increased levels of TFF peptides in conjunction with mucus protein indicate the formation of a protective gastric mucous barrier in the hsMPS, which enables the study of pathogenesis within the in vivo‐relevant gastric mucous barrier. Although a previous study revealed that gastric epithelial cells show effective mucin secretion when grown at the air–liquid interface (ALI), the effect of a TFF‐mediated chemical barrier has not been thoroughly investigated.^[^ ^9b^ ^]^ Adapting the ALI culture method to our hsMPS might be an interesting research opportunity to enhance the function of the gastric mucosal barrier.

### 2.5 Enhancing the Formation of Epithelial Junctional Complexes with gMSC Co‐Culture under Flow Conditions

**Figure 5:**
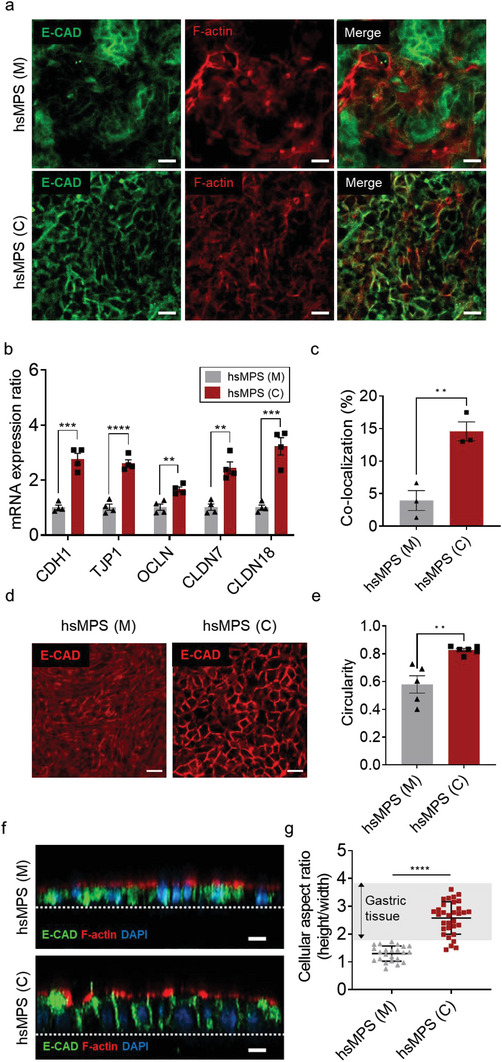
Epithelial junctional complexes in the hsMPS. a) Immunofluorescence micrographic images of gastric epithelium in the monocultures (M) and co‐cultures (C) in the hsMPS labeled with E‐cadherin (green) and F‐actin (red) (bar, 20 µm). b) mRNA expression ratio of genes encoding gastric epithelial junction proteins including E‐cadherin (*CDH1*), tight junction protein‐1 (*TJP1*), occludin (*OCLN*), claudin‐7 (*CLDN7*), and claudin‐18 (*CLDN18*) under co‐culture conditions relative to monoculture conditions in the hsMPS. c) Co‐localization efficiency of F‐actin and E‐cadherin analyzed using ImageJ. d) Representative immunofluorescence micrographic images of gastric epithelial cells stained with E‐cadherin (red) in monocultures (M) and co‐cultures (C) (bar, 20 µm). e) Circularity of gastric epithelial cells in monocultures and co‐cultures analyzed using ImageJ. f) Representative z‐stacking immunofluorescence images of gastric epithelial cells under monoculture and co‐culture conditions labeled with F‐actin (red) and E‐cadherin (green) (bar, 20 µm). g) Cellular aspect ratio of gastric epithelial cells in the monocultures (M) and co‐cultures (C) in the hsMPS. The results are presented as the mean ± SEM. For statistical analysis, a Student's *t*‐test was performed (*p < 0.05; **p < 0.01; ***p < 0.001; ****p < 0.0001).

The integrity of the gastric epithelium is tightly regulated by functional apical‐junctional complexes comprising tight and adherens junctions. Importantly, the adherens junction containing E‐cadherin plays a key regulatory role in epithelial barrier integrity and is highly associated with maturation and localization of secretory cells.^[^ ^30^ ^]^ To determine whether gMSCs contribute to the formation of junctional complexes in the hsMPS, we evaluated barrier integrity by observing E‐cadherin and F‐actin using a confocal immunofluorescence assay. As shown in **Figure** **5**a, the integrity of E‐cadherin in gastric epithelial cells was enhanced in hsMPS (C), which was confirmed using qRT‐PCR, which indicated a seven‐fold increase in mRNA expression of the CDH1 gene (Figure 5b). Furthermore, F‐actin was more concentrated in the cell–cell contact region, representing a higher co‐localization efficiency of F‐actin with E‐cadherin (4.0 ± 2.7% vs 15 ± 2.2%) (Figure 5a,c). As E‐cadherin stability depends on its link to the actin cytoskeleton,^[^ ^31^ ^]^ these data reveal that gMSCs facilitate the formation and structural integrity of the adherens junction. Moreover, the mRNA expressions of genes encoding other gastric tight junction proteins such as tight junction protein‐1 (*TJP1*), occludin (*OCLN*), claudin‐7 (*CLDN7*), and claudin‐18 (*CLDN18*) were significantly upregulated in hsMPS (C) compared to hsMPS (M) (Figure 5b).

During gastric tissue morphogenesis, increased intercellular adhesion along the lateral membrane and proliferation of epithelial cells in a confined area drive epithelial elongation.^[^ ^32^ ^]^ In the hsMPS, epithelial cells showed the typical cobblestone‐like phenotype of differentiated gastric epithelial cells when co‐cultured with gMSCs because E‐cadherin played a role in the maintenance of epithelial morphology and polarity^[^ ^33^ ^]^ (Figure 5a,e). Furthermore, 3D confocal microscopy of the hsMPS (C) revealed the formation of a simple columnar epithelium sheet composed of cells taller than they are wide (cell aspect ratio of approximately 2.5), as seen in in vivo gastric tissue^[^ ^34^ ^]^ (Figure 5f,g; Figure S4, Supporting Information). In contrast, in the hsMPS (M), gastric epithelial cells displayed spindle‐shaped morphology similar to a previous report^[^ ^6b^ ^]^ (Figure 5f,g; Figure S4, Supporting Information) and formed a flat epithelial sheet composed of cells with a lower cell aspect ratio (approximately 1.2). These results indicate the role of gMSCs in recapitulating the gastric epithelial barrier function and morphological features in the hsMPS by enhancing the junctional complexes and maintaining mitotic gastric stem cells under fluid flow.

### 2.6 The hsMPS Exhibits an Efficient TFF1‐Mediated Defense Mechanism against *H. pylori* Infection

In human gastric mucosa, *H. pylori* invasion of gastric epithelial cells is limited by the gastric mucus layer, which physically protects the epithelium, and TFF1 binds to *H. pylori* within gastric mucus for chemical protection. Due to the proinflammatory cytokines released from gastric epithelial cells, immune cells are recruited to the infected regions where they initiate the mucosal immune responses. However, *H. pylori* has its own strategies for evading the host defense system so as to survive and cause various gastric diseases.^[^ ^35^ ^]^ Hence, accurate approximation of gastric mucosal barrier functions at early stage provides a deep understanding of pathogen‐host interactions and development of treatment methods before malignant transformation of gastric tissue. We recapitulated early *H. pylori* pathogenesis and defense mechanisms in vitro using the hsMPS by inoculating *H. pylori* on the luminal compartment of hsMPS, which offers a physiologically relevant gastric mucosal barrier.

**Figure 6:**
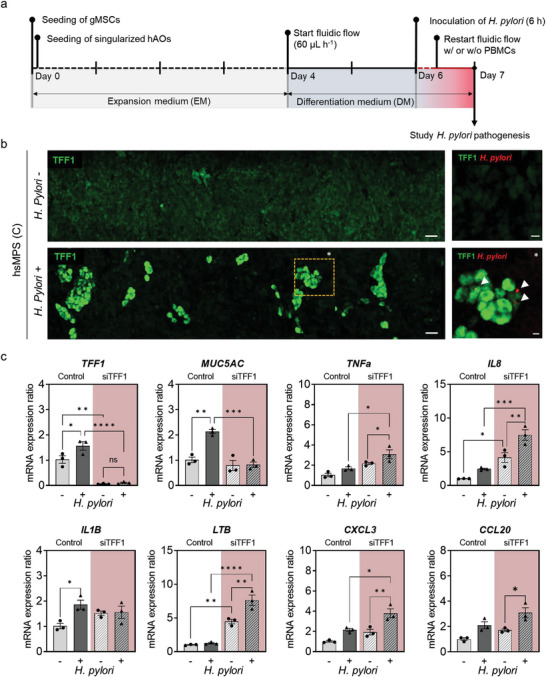
Protective function of the gastric mucosal barrier against *H. pylori* infection. a) Timeline for generation of the *H. pylori* infection model. At day 6, the gastric epithelium in the luminal microchannel of hsMPS is infected with *H. pylori* for 6 h under static conditions and fresh DM is supplied at 60 µL h^−1^ for another 18 h. b) Low magnification (left; bar, 50 µm) immunofluorescence micrographic images of the human gastric epithelium labeled with TFF1 (green) of a noninfected (top) and an *H. pylori*‐infected hsMPS at an MOI of 10 (bottom). High magnification (right; bar, 20 µm) immunofluorescence micrographic images showing gastric epithelium labeled with TFF1 (green) and *H. pylori* (red). c) The mRNA expression ratios of NF‐κB‐mediated inflammatory cytokines *TNFa*, *IL8*, *IL1B*, *LTB*, *CXCL3*, and *CCL20*, and mucus‐associated proteins *TFF1* and *MUC5AC*, in gastric epithelial cells cultured in hsMPS (C) where TFF1 was knocked down using siRNA‐TFF1 (siTFF1) and infected with *H. pylori* compared with controls infected with or without *H. pylori*. The results are presented as the mean ± SEM. For statistical analysis, one‐way ANOVA and Tukey's test was performed (*p < 0.05; **p < 0.01; ***p < 0.001; ****p < 0.0001). MOI, Multiplicity of infection. NI, non‐infected.

Many studies have confirmed that TFF1 is upregulated in response to inflammation to protect gastric epithelium through autocrine and paracrine anti‐inflammatory functions.^[^ ^36^ ^]^ Thus, the pattern of TFF1 expression and localization of *H. pylori* was monitored via confocal immunofluorescence after co‐culturing our hsMPS with *H. pylori* strain J99 expressing cagA‐ and vacA‐positive genotype. Because histopathological studies indicate that an multiplicity of infection (MOI) of approximately 10 generally appears in *H. pylori*‐colonized gastric mucosa,^[^ ^37^ ^]^ an MOI of 10 was used to study the response of hsMPS to *H. pylori* (**Figure** **6**a). Interestingly, the expression of TFF1 was highly upregulated in distinct regions of the gastric epithelium in the vicinity of the *H. pylori* colonization zone, 24 h after inoculation (Figure 6b). While it remains to be investigated further, it is reasonable to assume that TFF1 biological fence was formed via paracrine action of epithelial cells infected with *H. pylori*. It may induce TFF1 expressions in nearby cells to intensively protect the epithelium from the spreading of *H. pylori*. Further studies are required to better understand the underlying molecular mechanisms. On the other hand, the hsMPS (M) secreted lower levels of TFF1 and did not show the formation of TFF1 islands when infected with *H. pylori*, which resulted in less efficient control of the expansion of *H. pylori*, thereby leading to greater coverage of the epithelial layer by *H. pylori* (Figure S5, Supporting Information). This was also observed when culturing an AGS cell line in an MPS device, which is known to express very low TFF1 and MUC5AC levels compared with hAOs, which showed *H. pylori* spread to the entire AGS cell layer (Figure S5, Supporting Information). The root mean square of the fluorescent intensity of TFF1 in hsMPS (C) infected with *H. pylori* was significantly greater than that in non‐infected group, suggesting that *H. pylori* infection caused a localized increase in the expression of TFF1 (Figure S6, Supporting Information). In addition, we found that TFF1 biological barrier disappears in hsMPS (C) four days after being infected with *H. pylori* at an MOI value of 10. These findings align with earlier research showing that TFF1 expression is initially elevated during acute *H. pylori* infection in vivo but declines during chronic infection (Figure S7, Supporting Information).^[^ ^38^ ^]^

To our knowledge, this is the first demonstration of patterned TFF1 expression to defend against *H. pylori*. This cannot be found using global gene expression analysis conducted in past studies and is associated with the chemical defense mechanism of the gastric mucosal barrier in efficiently blocking the spread of *H. pylori* within fenced regions. This finding might help to understand TFF1‐mediated *H. pylori* defense mechanisms, which is less studied because of the lack of physiologically relevant in vitro models.

Furthermore, the role of TFF1 in modulating inflammation during *H. pylori* infection was investigated in our stomach systems (Figure 6c). To examine the TFF1‐mediated modulation of inflammation hsMPSs (C), gastric epithelial cells were treated with siRNA‐TFF1 (siTFF1) to inhibit the expression of TFF1 before inoculation with *H. pylori*. The downregulation of TFF1 in hsMPS (C) was confirmed using qRT‐PCR analysis. At 24 h after inoculation with *H. pylori*, we observed the change in expression levels of TFF1 and MUC5AC, as well as the transcription factor nuclear factor kappa B (NF‐κB) signaling pathway, which is a known pathophysiological feature of *H. pylori* infection. Specifically, we observed the expression of proinflammatory cytokines encoding tumor necrosis factor alpha (*TNFa*), IL‐8 (*IL8*), IL‐1 beta (*IL1B*), lymphotoxin beta (*LTB*), and CC/CXC motif chemokine ligands (*CXCL3* and *CCL20*) (Figure 6c), which were previously found to be overexpressed in patients infected with *H. pylori*.^[^ ^39^ ^]^

The gastric epithelial cells in control hsMPS (C) showed significant increase in the level of TFF1 and MUC5AC at 24 h after inoculation with *H. pylori*. Conversely, the hsMPS (C) in which TFF1 expression was inhibited did not show any change in TFF1 or MUC5AC when infected with *H. pylori*. They also exhibited upregulated inflammatory gene expressions (*TNFa*, *IL8*, *LTB*, *CXCL3*, and *CCL20*) (Figure 6c), confirming the mucosal protective functions of TFF1 that counteracts the development of severe inflammation by offering binding site for *H. pylori*. This implies that hsMPS (C) having a well‐developed mucosal barrier can recreate how bacteria colonization and further development of severe inflammation are prevented. Previous studies showed that TFF1 knock‐out mice models exhibit high‐grade inflammation scores, resulting in a higher risk of gastric cancer when infected with *H. pylori*.^[^ ^40^ ^]^ Therefore, our results demonstrate the importance of TFF1 in modulating inflammation during *H. pylori* infection in our stomach systems.

### 2.7 Complex *H. pylori*‐Induced Cellular Responses in the hsMPS Co‐Cultured with gMSCs

Based on the demonstration of better physiological relevance of hsMPS (C) compared with hsMPS (M) or hAOs, we focused on monitoring *H. pylori* infection‐mediated cellular responses in the hsMPS (C) at different MOI values. First, we analyzed mRNA expression of genes encoding TFF1, TFF2, and MUC5AC after infection with *H. pylori* at different MOI values ranging from 0.1 to 200 (Figure S8, Supporting Information). The mRNA expression of TFF1 was significantly upregulated in *H. pylori*‐positive hsMPS compared with that in the non‐infected group. However, this upregulation was slightly lower at an MOI value of 200 compared with the upregulation at MOI values of 0.1 and 10. It was also revealed that mRNA expression levels of TFF2 and MUC5AC were significantly increased in the *H. pylori‐*positive sample, but not in an MOI‐dependent manner (Figure S8, Supporting Information). This low dependence on the MOI (Figure S8, Supporting Information) may explain a limited protective role of mucosal defense system when scale of *H. pylori* colonization is highly increased in the host stomach. Past studies have shown that TFF1, TFF2, and MUC5AC expressions are initially upregulated in acute *H. pylori* infection in vivo, while they are decreased in chronic infection,^[^ ^38^ ^]^ implying that our model reproduced the protective mechanism of the gastric mucosal barrier against *H. pylori* when contacted with *H. pylori* at early stage. The mRNA expressions of gastric junctional proteins including *TJP1*, *OCLN*, and *CLDN18* were decreased when infected with *H. pylori* at an MOI value of 200, indicating the disruption of gastric barrier function in high MOI infection (Figure S9, Supporting Information). Furthermore, the mRNA expressions of *TNFa*, *IL8*, *IL1B*, *LTB*, *CXCL3*, and *CCL20* were significantly upregulated at an MOI of 200. However, at an MOI of 10, although there was a trend of increased inflammatory genes in the epithelial cells, most of these genes were not significantly upregulated. This finding indicates that the protective role of the mucosal defense system becomes limited when the scale of *H. pylori* colonization significantly increases in the host stomach (Figure S10, Supporting Information).

**Figure 7:**
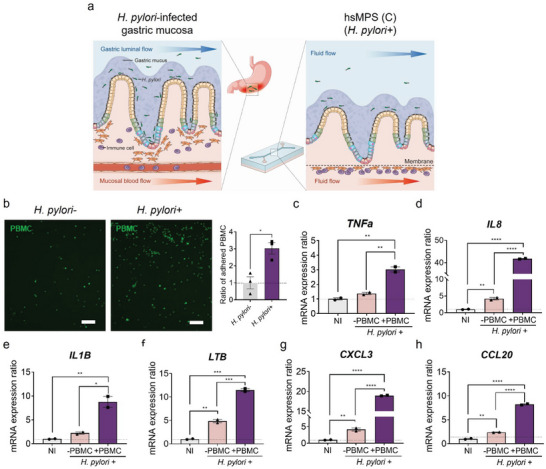
Immune pathogenesis in *H. pylori*‐infected hsMPSs co‐cultivated with PBMC. a) Schematic illustration of the *H. pylori*‐infected human gastric mucosa (left) and *H. pylori*‐infected hsMPSs co‐cultured with peripheral blood mononuclear cells (PBMCs). b) Representative fluorescence images of PBMCs adhering to the basal side of the abluminal microchannel membrane of a non‐infected (upper, left) and *H. pylori*‐infected hsMPS at an MOI of 10 (lower, left) stained with CellTracker Green (bar, 250 µm). Relative number of PBMCs adhering to the basal side of the membrane of *H. pylori*‐infected hsMPS at MOI 10 compared to non‐infected control (right). For statistical analysis, a Student's *t*‐test was performed (*p < 0.05). The mRNA expression ratios of NF‐κB‐mediated inflammatory cytokines c) *TNFa*, d) *IL8*, e) *IL1B*, f) *LTB*, g) *CXCL3*, and h) *CCL20* in gastric epithelial cells when infected with *H. pylori* at an MOI of 10 in the presence and absence of PBMCs in the abluminal microchannel relative to the non‐infected control (NI). The results are presented as the mean ± SEM, *n*  =  2 for the independent hsMPS experiments. For statistical analysis, one‐way ANOVA with Tukey's multiple comparisons test was performed (*p < 0.05; **p < 0.01; ***p <0.001; ****p < 0.0001). TNFa, tumor necrosis factor alpha; IL, interleukin; LTB, lymphotoxin beta; CCL/ CXCL, chemokine (C‐C/C‐X‐C motif) ligand; −, without PBMC; +, with PBMC.

As pro‐inflammatory cytokines and chemokine ligands were increased in hsMPSs infected with *H. pylori* with activation of NF‐κB signaling, we investigated whether our model could recapitulate the chemotaxis of immune cells shown in *H. pylori*‐infected gastric tissue, which may lead to further chronic active inflammation in *H. pylori* infection (**Figure** **7**a). Human primary PBMCs (peripheral blood mononuclear cells) were introduced in the abluminal channel, while *H. pylori* (MOI 10) was introduced into the luminal channel in the flow system (Figure 7a). As expected, the number of PBMCs adhering to the abluminal side of the membrane was approximately 2.5‐fold higher in the hsMPS infected with *H. pylori* than that in the non‐infected hsMPS, emulating immune cell recruitment at the *H. pylori* infection site (Figure 7b). Because of the interplay between gastric epithelium and immune cells, the hsMPS in combination with PBMC, showed a greatly enhanced response level in terms of the upregulation of NF‐κB and cytokine expression (Figure 7c–h), as observed in vivo. In particular, IL‐8 that is involved in all significant response pathways in the initial cellular response to *H. pylori* infection^[^ ^37^ ^]^ (Figure 7d), and CXCL3 (Figure 7g) and CCL20 (Figure 7h), important neutrophil chemo‐attractants,^[^ ^41^ ^]^ were highly activated in the presence of the immune cells, thus showing the contribution of immune cells in initial pathogenic mechanisms of *H. pylori* infection. These studies demonstrated the potential of the hsMPS as a promising in vitro tool to study immune pathogenic mechanisms of *H. pylori* infection, and to further identify physiological contributions of individual immune cell types.

## 3 Conclusion

The utilization of hAOs has effectively addressed the limitations associated with neoplastic or cancerous gastric epithelial cell lines, allowing for the integration of multiple types of gastric epithelial cells and enabling the manifestation of complex gastric functions.^[^ ^42^ ^]^ In this study, exploiting MPS technology facilitated dynamic cell culture through co‐cultivation in a separate channel or controlled fluid flow, resulting in a more sophisticated microenvironment control for maintaining gastric epithelial homeostasis. Specifically, the maintenance of mesenchymal niche function of gMSCs through fluid flow played a crucial role in supporting the physiological transit‐amplifying process, which is essential for maintaining a balance between gastric epithelial proliferation and differentiation.

The functionalities provided by the system enhanced the formation of epithelial junctional complexes, establishing an epithelial barrier and replicating an in vivo‐like continuous mucus barrier over the gastric epithelium with high expression levels of TFF1 and TFF2. Consequently, the system successfully mimicked infection‐induced gastric epithelial responses. Compared to enclosed hAOs,^[^ ^43^ ^]^ the hsMPS demonstrated improved efficiency in the inoculation of *H. pylori* to the apical side of the epithelium, while the excretion of metabolic wastes allowed for a more accurate approximation of cellular responses to pathogens. Furthermore, the two‐channel MPS device not only facilitated the individual acquisition and analysis of cells from each channel but also enabled accurate analysis of secreted factors from different channels. Our hsMPS represents a significant advancement as the first stomach MPS capable of recapitulating the in vivo environment of the gastric mucosal barrier. As a result, it will enhance drug development and therapeutic approaches for gastric bacterial and viral infections. One limitation of our hsMPS is the undesired absorption of small molecules in a device, due to the intrinsically highly porous nature of PDMS, which can pose challenges in accurate prediction of drug toxicity and efficacy.^[^ ^44^ ^]^ To ensure reliable therapeutic testing against pathogenic infections, it is crucial to explore the development of MPS devices using alternative materials that mitigate the issue of molecule absorption.

This study only focused on the recapitulation of the gastric mucosal barrier in the antrum region to study initial *H. pylori* infection and immune responses. However, gastric physiology and pathophysiology rely on the complex interplay between the antrum and corpus regions of the stomach. In the stomach, gastric acid, which determines the pH of gastric juice, is secreted from parietal cells in the corpus region.^[^ ^45^ ^]^ Gastric acid secretion in the corpus region is mediated by hormonal agents, including gastrin secreted from the antrum, as well as paracrine and neuronal pathways.^[^ ^45^ ^]^ Thus, a limitation of the current research is that intragastric pH was not appropriately recapitulated in our hsMPS, which only contained antrum stem cell‐derived epithelial cells. The gastric pH can be also affected by pathogens such as *H. pylori* when spreading from the antrum mucosa to the corpus and inducing atrophy, hypergastrinemia, or gastric adenocarcinoma.^[^ ^46^ ^]^ Therefore, further work on developing MPS platforms which offer functionally linked antrum and corpus regions would help us comprehensively understand chronic *H. pylori* pathogenesis involving entrance, penetration, and cagA translocation of *H. pylori* in the pH gradient environment and investigate potential pharmacological approaches to gastric acid suppression for the treatment of acid‐related disorders. The presence of a larger population of gastrin‐secreting cells in our stomach system, which play a role in regulating gastric acid secretion by parietal cells in the corpus region, highlights the potential for developing an antrum‐corpus linked model (Figure S1, Supporting Information). In addition, to gain a comprehensive understanding of the epithelial‐mesenchymal interactions in gastric epithelial homeostasis, it is necessary to elucidate the molecular and functional characteristics of activated gMSCs in the hsMPSs. Furthermore, studying the signaling pathways involved, such as Wnt, Hedgehog,^[^ ^5^ ^]^ and BMP,^[^ ^47^ ^]^ is essential.

## 4 Experimental Section

### Generation of hAOs and Gastric Mesenchymal Stromal Cell Cultures

Human antrum was collected from patients who underwent surgery at the Seoul National University College of Medicine (IRB protocol number: H‐2002‐082‐1102). The hAOs were generated as previously described^[^ ^48^ ^]^ with some minor modifications. The antrum tissue was washed in Dulbecco's phosphate‐buffered saline (DPBS) with antibiotics (Primocin and Plasmocin, InvivoGen) and was pinned on a silicone‐coated Petri dish. The mucosa was carefully separated from the submucosa under a microscope using microdissecting scissors and washed in ice‐cold chelating buffer (DPBS without Ca^2+^/Mg^2+^ containing 1% sucrose, 2% D‐sorbitol and 1% bovine serum albumin). The surface of the mucosa was gently scraped using curved forceps to remove the gastric mucus. The mucosa was cut into small pieces (1–2 mm^2^) and incubated in 10 mm EDTA‐chelating buffer on a shaker for 10 min at room temperature (RT). Tissue fragments were washed with ice‐cold chelating buffer and covered with a glass slide on a petri dish. The gastric glands were isolated by applying pressure to the glass slide and directly suspended in advanced DMEM/F12 (Gibco) with 5% fetal bovine serum (FBS) in a conical tube. After centrifugation for 5 min at 250 × *g* at 4 °C, the pellet was resuspended in 20 µL Matrigel and cultured in a 48‐well cell culture plate in the EM (Table S1, Supporting Information). The hAOs were passaged twice a week with a split ratio of 1:3 to 1:6.

The gMSC culture was established using gastric muscularis mucosae harvested from the gastric antrum tissue. The gastric muscularis mucosae were cut into small fragments (2–5 mm^2^), washed in DPBS, and then placed in a buffer containing type I collagenase (Sigma) and Accutase (Merck) for 1.5 h in a shaker at 37 °C. The digest was subsequently treated with 5% FBS (Merck) to inactivate the enzymes. The dissociated cells and tissue fragments were centrifuged at 1000 rpm for 5 min at 4 °C and cultured in a T75 tissue‐culture flask in growth medium (advanced DMEM/F12 [Gibco] containing 5% bovine serum [Gibco]). The gMSCs were allowed to migrate from the tissue fragments attached to the dish and form a monolayer. The cells were cultured for approximately 7 days until they reached 80% confluency and then passaged for establishing a gMSC line.

### Device Fabrication

The design of the hsMPS was modified from a previously reported human BBB‐on‐a‐chip.^[^ ^49^ ^]^ The apical and basal channels were cast in polydimethylsiloxane (PDMS) (Sylgard 184) at a 10:1 ratio of base to curing agent in a custom acryl‐patterned master mold. The microchannel was 2 cm long and 1 mm wide. The top and bottom channels were 1 and 0.2 mm high, respectively. After curing the PDMS at 60 °C for 6 h, the PDMS replica was demolded from the master mold. Track‐etched polyethylene terephthalate (PET) membranes (1 µm pore size) purchased from it4ip were bonded to PDMS, as previously described. The PDMS parts and PET membranes were treated with oxygen plasma (80 sccm O_2_ for 1 min). The PDMS parts were submerged in a 5% 3‐aminopropyltriethoxysilane (Sigma) solution to generate surface amine, and PET membranes were submerged in a 1% 3‐glycidoxypropyltriethoxysilane (Sigma) solution for epoxy functionalization for 20 min at RT. Parts were rinsed in water and dried with compressed air. The microfluidic device was assembled by placing a PET membrane between the PDMS parts and aligning them with the microchannel pattern. The assembled devices were placed in a 65 °C oven for 2 days to form a strong amine‐epoxy bond.

### Reconstitution of the hsMPS

The PDMS surface of the chip was activated by treating with plasma (30 sccm air, for 1 min), and coated with 1% Matrigel for 1.5 h at 37 °C. For co‐culturing the gMSCs in a chip, gMSCs at a density of 1.25 × 10^6^ cells mL^−1^ were seeded on the abluminal channel in a gMSC medium. The chip was flipped immediately to allow the gMSCs to attach to the Matrigel‐coated PET membrane and then placed in the CO_2_ incubator. Two hours after seeding the gMSCs, the chip was flipped back, and the apical and basal channels were rinsed with EM. Gastric antrum epithelial cells were prepared by singularization of the hAOs by treatment with TrypLE express (Gibco) for 5 min at 37 °C. Gastric antral epithelial cells at a density of 5 × 10^6^ cells mL^−1^ were seeded on the luminal channel in EM. After 24 h, the hsMPS was fed with fresh EM every day for an additional 3 days. On day 4 of cell seeding, the outlet channels of the chip were connected to syringe pumps (Fusion 200, Chemyx Inc.) and a DM (Table S1, Supporting Information) was flowed through the channels at 60 µL h^−1^. For static hsMPS culture, the medium was switched to DM on day 4 and replaced daily with fresh DM to maintain the cells.

In order to assess whether the activation of Notch signaling improved the cellular niche functions of gMSCs, a 100 nm Notch inhibitor (LY3039478, Selleckchem) was administered to the abluminal channel under fluid flow conditions with DM for 2 days. To measure the quantity of paracrine molecules released by gMSCs cultured under both static and fluid flow conditions for maintaining gastric stem cell homeostasis, protein‐specific ELISA kits (listed in Table S2, Supporting Information) were employed and the manufacturer's protocol was followed for each kit.

### Quantitative Real‐Time PCR

The mRNA expression levels were analyzed with quantitative real‐time polymerase chain reaction (qRT‐PCR). Total RNA of cells within a microchannel was extracted using a RNeasy Kit (Qiagen) and cDNA was synthesized using a QuantiTech Reverse Transcription Kit (Qiagen) following the manufacturer's protocol. The qRT‐PCR was carried out using a SYBR Green Realtime PCR Master Mix (TOYOBO) in a CFX Connect Real‐Time PCR Detection System (Bio‐Rad). The forward and reverse primers used for qRT‐PCR are provided in Table S3, Supporting Information.

### Modeling *H. pylori* Infection

*H. pylori* strain J99 (American Type Culture Collection 700824), expressing virulence factors such as cagA‐positive and vacA‐positive genotype, was grown following the manufacturer's protocol. Prior to inoculation of *H. pylori* into the chip, *H. pylori* was washed three times with DPBS and centrifuged for 3 min at 6000 × *g* at 4 °C. To avoid contamination within the abluminal channel, both the inlet and outlet of the abluminal channel were blocked with yellow tips. *H. pylori* was then gently inoculated into the luminal channel after suspending *H. pylori* with the antibiotics‐free DM. Six hours after inoculation, the microfluidic device was connected to the chip and fresh culture media was provided to both microchannels at 60 µL h^−1^ for 24 h.

To verify the protective effect of TFF1 peptides, functional inhibition was conducted by interfering with mRNA expression. To suppress the expression of the target TFF1 mRNA level, gastric epithelial cells were transfected with a pre‐designed small interfering RNA (siRNA, Sense; GGAGUGUGAAUUUUAGACAtt, Antisense; UGUCUAAAAUUCACACUCCtc, Thermo Fisher) using Lipofectamine RNAiMAX Transfection Reagent (Invitrogen) on culturing day 4 before the differentiation period for 12 h.

To co‐cultivate the *H. pylori* infection model with PBMCs, PBMCs were collected from peripheral blood of healthy donors (IRB protocol number: IRB‐20‐44‐A) using Lymphoprep Density Gradient Medium (Stemcell Technologies Inc.) following the manufacturer's protocol. Before adding PBMCs to the abluminal microchannel, PBMCs were stained using 5 µm of CellTracker Green (Invitrogen) following the manufacturer's protocol. The dyed PBMCs (1 × 10^6^ cells) were flowed into the abluminal channel after a 6 h inoculation.

### Immunofluorescence Microscopic Analysis

The hsMPS was fixed with 4% paraformaldehyde in DPBS for 15 min and washed with DPBS three times for 5 min. Immunostaining was performed after permeabilization in DPBS with 0.1% Triton X‐100 (Sigma) and blocking for 1 h in 10% goat serum in DPBS with 0.1% Triton X‐100. The hsMPS microfluidic channels were treated with primary antibodies (Table S4, Supporting Information) and incubated overnight at 4 °C. After the washing procedure, secondary antibodies (Table S4, Supporting Information) were incubated for 1 h at RT. Nuclei were counterstained with 4′,6‐diamidino‐2‐phenylindole (DAPI; Sigma). The human gastric antral tissues were also fixed with 4% paraformaldehyde in DPBS for overnight at 4 °C and washed DPBS three times. The fixed tissues were dehydrated in a Tissue processor (Thermo Scientific Microm) and embedded in paraffin using paraffin embedding center (Leica Microsystems). The paraffin‐embedded tissue blocks were sectioned into 7 µm section with Microtome (Leica Microsystems). These section tissues were mounted on coated slides. These slide samples were stained with each antibody after deparaffinization (xylene for 3 min), rehydration (100%, 95%, 70% ethanol), antigen retrieval (antigen unmasking solution for 5 min using pressure cooker) and blocking (5% bovine serum albumin) steps. Conventional confocal imaging was carried out using confocal microscopes LSM780NLO (Zeiss) and LSM980 (Zeiss).

To compare the gastric epithelial cell morphology of monoculture and co‐culture, the area, perimeter, width, and height of each cell was measured by using ImageJ software with immunofluorescence images of E‐cadherin. Using each cell area and perimeter, the circularity could be calculated with the following formula:

(1) $$\mathrm{Circularity}=4\pi \frac{\mathrm{Cell}\;\mathrm{area}}{\mathrm{Cell}\;\mathrm{perimeter}}$$

Additionally, the cellular aspect ratio was calculated by the following formula:

(2) $$\mathrm{Cellular}\;\mathrm{aspect}\;\mathrm{ratio}=\frac{\mathrm{Cell}\;\mathrm{width}}{\mathrm{Cell}\;\mathrm{height}}$$

To confirm the correlation between *H. pylori* and TFF1 island formation, the fluorescent intensity of TFF1 in each pixel in the confocal immunofluorescence images of hsMPS (C) in non‐infected and *H. pylori*‐infected conditions was analyzed, and further the root mean square (RMS)^[^ ^50^ ^]^ of fluorescent intensity was calculated, which indicated the pixel intensity deviation from the mean value.

(3) $$\mathrm{RMS}x=\sqrt{\frac{1}{n}\left(x_{1}^{2}+x_{2}^{2}+\cdots x_{n}^{2}\right)}$$

where *x_n_* indicates the deviation of individual data point (fluorescent intensity) from the mean.

### Flow Cytometric Analysis

Harvested cells from hAO or hsMPS were singularized using TrypLE express (Gibco) for 10 min, and dissociated cells were fixed with 4% PFA for 30 min at RT. Cells were permeabilized with an antibody dilution buffer (DPBS containing 0.1 vol% Triton X‐100 and 2 wt% bovine serum albumin) for 30 min at RT. Afterward, cells were spun down for 3 min at 300 × *g* at 4 °C. The cell pellet was resuspended in the antibody dilution buffer containing primary antibodies (Table S3, Supporting Information) and incubated for 1 h on ice in the dark. Following incubation, the cells were washed twice with the antibody dilution buffer, and incubated with fluorescent‐conjugated secondary antibodies (Table S3, Supporting Information) in the same buffer for 30 min on ice in the dark. The cell suspension was washed twice, and the labeled cells were resuspended in 200 µL of the antibody dilution buffer. The labeled cell suspension was transferred to an Ultra‐Low Attachment (ULA) 96‐well plate (Corning), which was then loaded onto a Novocyte instrument (Agilent Technologies) for flow cytometry analysis.

### Statistical Analysis

All data represent means (±SEM). Statistical analyses were performed with GraphPad Prism 8 (GraphPad Software), using a Student's *t*‐test to compare two data sets, and one‐way analysis of variance (ANOVA) with Tukey's multiple comparisons test to compare multiple groups (*p < 0.05; **p < 0.01; ***p < 0.001; ****p < 0.0001).

### Ethical Approval

This study involves human participants and the protocol was reviewed and approved by the institutional review board (IRB: H‐2002‐082‐1102 and IRB‐20‐44‐A). Participants gave informed consent to participate in the study.

## Conflict of Interest

The authors declare no conflict of interest.

## Author Contributions

H.‐J.J. participated in the design and performance of all experiments and analyzed the data with T.‐E.P. and S.‐H.K., who supervised all work. J.‐H.P. and S.‐H.K. obtained gastric tissue samples according to the IRB‐approved protocol. J.K. provided scientific supervision of the *H. pylori* infection experiments. J.S.D. participated in the analysis of image data. H.‐J.J. and T.‐E.P. prepared the manuscript with input from all others.

## Supporting information

Supporting Information

## Data Availability

The data that support the findings of this study are available in the supplementary material of this article.
