# Supplementary material for: Organoid‐Based Human Stomach Micro‐Physiological System to Recapitulate the Dynamic Mucosal Defense Mechanism
Source: Adv Sci (Weinh). 2023 Jul 31;10(27):2300164. doi: 10.1002/advs.202300164 (PMC10520631; doi:10.1002/advs.202300164)
Supplement: Supplementary file 1 — Supporting Information [file ADVS-10-2300164-s001.pdf]

ADVANCED SCIENCE
Open Access

Supporting Information

for *Adv. Sci.*, DOI 10.1002/advs.202300164

Organoid-Based Human Stomach Micro-Physiological System to Recapitulate the Dynamic Mucosal Defense Mechanism

Hye-Jin Jeong, Ji-Hyeon Park, Joo H. Kang, Jonathan Sabaté del Río, Seong-Ho Kong and Tae-Eun Park**

Supplementary Information

Organoid-Based Human Stomach Micro-Physiological System to Recapitulate the Dynamic Mucosal Defense Mechanism

Hye-Jin Jeong[1], *Ji-Hyeon Park*[2,4], *Joo H. Kang*[1], *Jonathan Sabaté del Río*[3], *Seong-Ho Kong*[2,*], *Tae-Eun Park*[1,*]

[1]Department of Biomedical Engineering, Ulsan National Institute of Science and Technology, Ulsan, 44919, Republic of Korea

[2]Department of Surgery, Seoul National University Hospital, Seoul National University College of Medicine, Seoul, 03080, Republic of Korea

[3]Center for Soft and Living Matter, Institute for Basic Science (IBS), Ulsan, 44919, Republic of Korea

[4]Department of Surgery, Gachon University Gil Medical Center, Incheon, 21565, Republic of Korea

*Corresponding Authors:
Seong-Ho Kong, MD, PhD (E-mail: seongho.kong@snu.ac.kr)
Tae-Eun Park, PhD. (E-mail: tepark@unist.ac.kr)

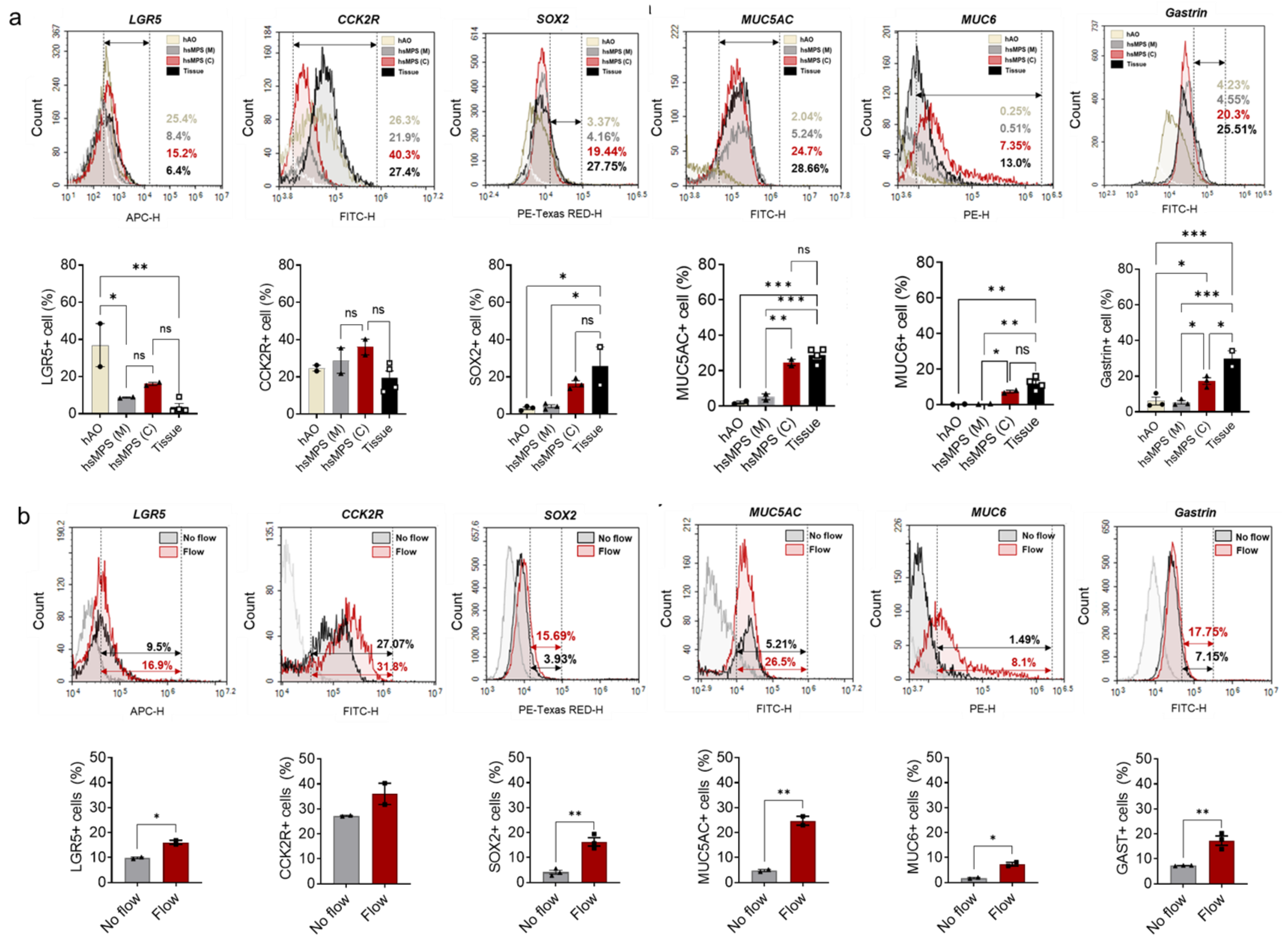

Supplementary Figure S1. Flow cytometry analysis of LGR5+, CCK2R+, SOX2+, MUC5AC+, MUC6+, Gastrin+ cells in gastric epithelial cells in the hsMPS (C) (a) compared to hAOs, hsMPS (M), and human tissue and (b) under static versus flow conditions. The results are presented as the mean ± SEM. For statistical analysis, a Student's *t*-test was performed (*$P < 0.05$; **$P < 0.01$; ***$P < 0.001$; ****$P < 0.0001$).

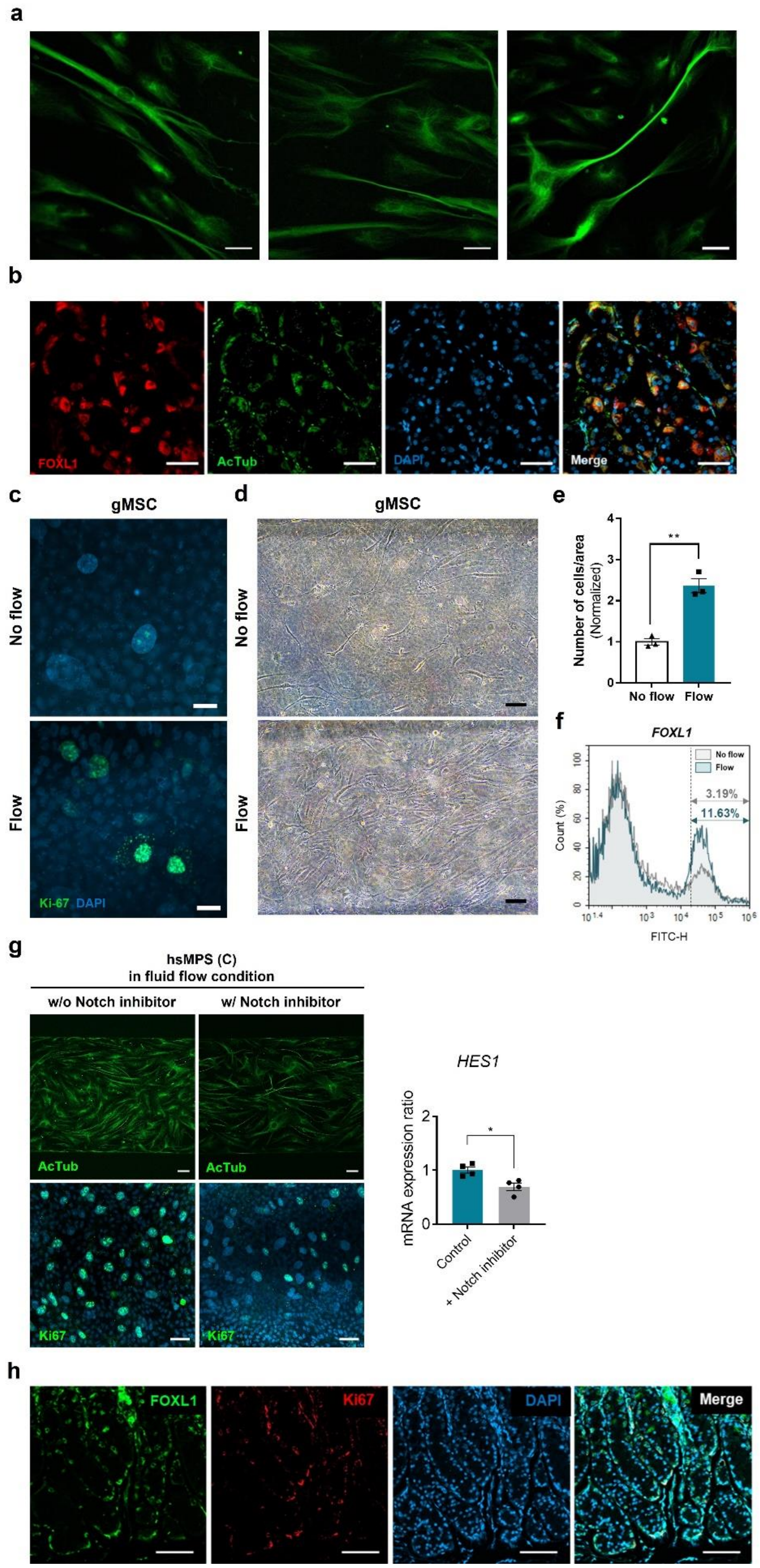
a
b
FOXL1
AcTub
DAPI
Merge
c
gMSC
No flow
Flow
Ki-67 DAPI
d
gMSC
No flow
Flow
e
Number of cells/area (Normalized)
**
No flow
Flow
f
FOXL1
No flow
Flow
3.19%
11.63%
Count (%)
FITC-H
g
hsMPS (C) in fluid flow condition
w/o Notch inhibitor
w/ Notch inhibitor
AcTub
Ki67
HES1
mRNA expression ratio
*
Control
+ Notch inhibitor
h
FOXL1
Ki67
DAPI
Merge

Supplementary Figure S2. Mitotic activity of gastric epithelium and mesenchymal stromal cells (gMSCs) in the hsMPS. (a) Confocal immunofluorescence images of gMSCs labeled with acetylated tubulin (AcTub, green) under flow conditions (bar, 50 μm). (b) Confocal microscopic analysis of gastric antral tissue labeled with Foxl1, AcTub, and DAPI (Scale bar, 50 μm). (c) Immunofluorescence images of Ki-67 in gMSCs (bar, 20 μm) under static (top) and flow conditions (bottom). (d) Bright field image of gMSCs in the basal channel under static (top) and flow conditions (bottom) (bar, 100 μm). (e) Ratio of number of gMSCs per area under static (top) and flow conditions (bottom). (f) Flow cytometry analysis of FOXL1+ cells in gMSCs in flow conditions compared to static conditions. (g) Notch inhibition in gMSCs by treatment with LY3039478 in abluminal channel of the hsMPS (C). Confocal immunofluorescence analysis of gMSCs treated with LY3039478, an inhibitor of Notch signaling (left). mRNA expression ratios of *HES1* in gMSCs (right). (h) Confocal microscopic analysis of gastric antral tissue labeled with Foxl1, Ki67, and DAPI (Scale bar, 50 μm). The results are presented as the mean ± SEM. For statistical analysis, a Student's *t*-test was performed (*$P < 0.05$; **$P < 0.01$; ***$P < 0.001$; ****$P < 0.0001$).

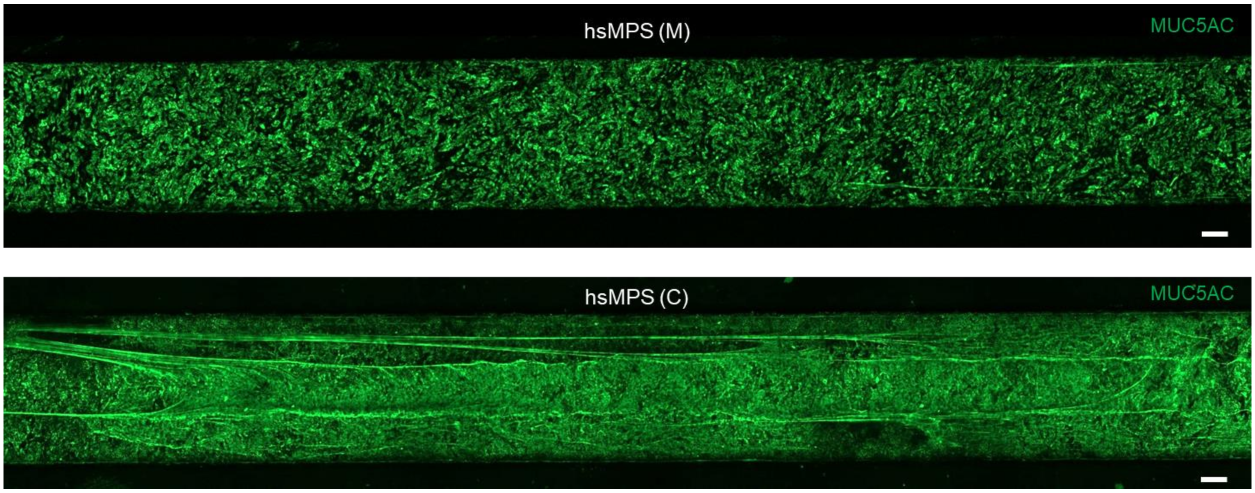

Supplementary Figure S3. Stitched tile-scanned immunofluorescence micrograph of gastric epithelium labeled with mucin 5AC (green) under monoculture (M) and co-culture (C) conditions. (bar, 200 μm).

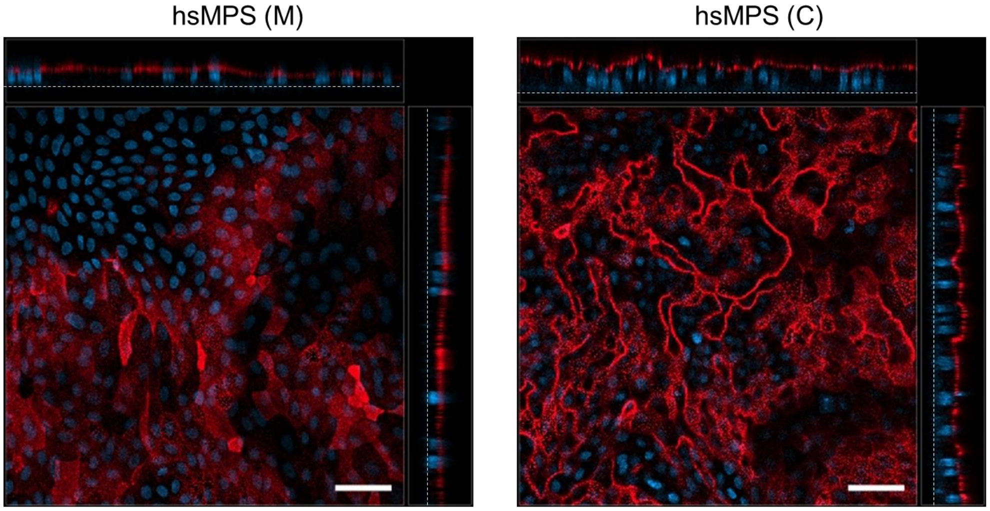

Supplementary Figure S4. Immunofluorescence confocal images of gastric epithelial cells labeled with F-actin (red) in the hsMPS device showing buckling of the epithelial layer when co-cultured with gMSCs under flow conditions (bar, 20 μm).

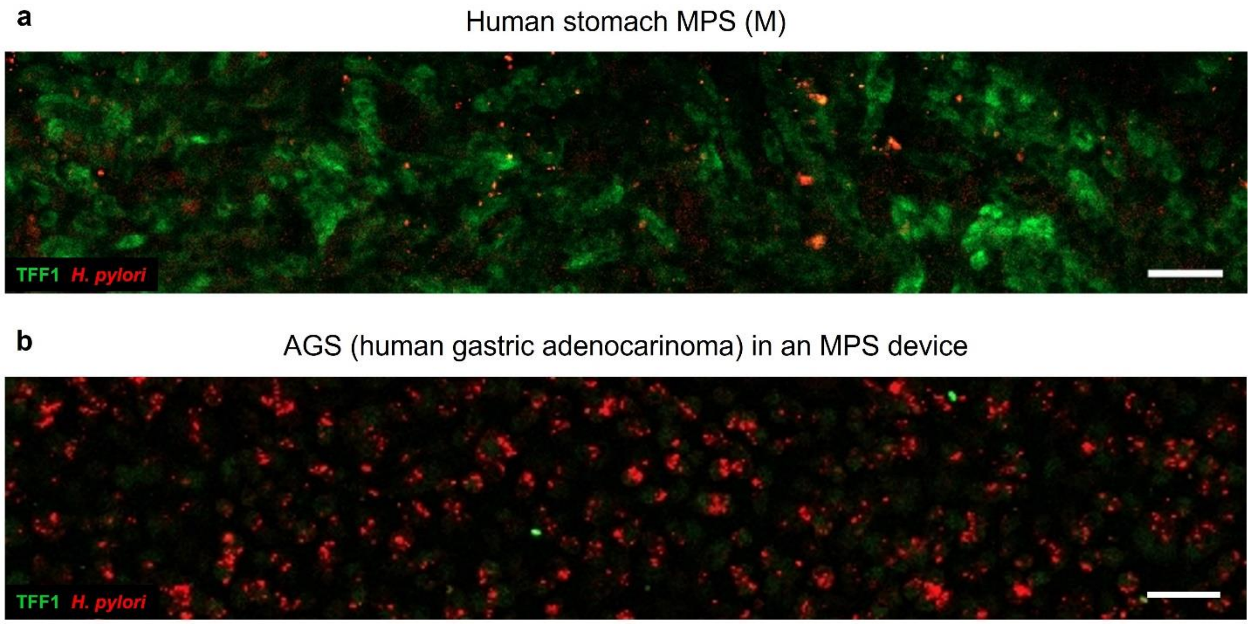

Supplementary Figure S5. *H. pylori*-infected gastric epithelium labeled with TFF1 and *H. pylori* in the hsMPS (M) and AGS cells cultured in an MPS device. Immunofluorescence micrographic images of the human gastric epithelium labeled with TFF1 (green) and a *H. pylori* (red) of mono-cultures (M) (a) and AGS cells (b) in an MPS device at an MOI 10. (bar, 50 μm). MOI, Multiplicity of infection.

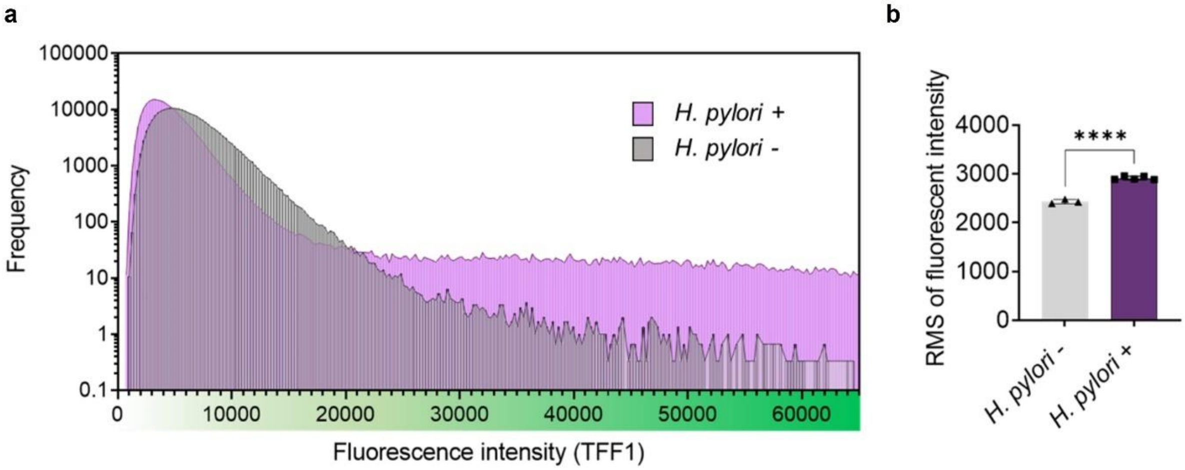

Supplementary Figure S6. (a) Frequency of each pixel corresponding to the fluorescent intensity of TFF1 in the hsMPS (C) in two conditions (*H. pylori* infected and non-infected). (b) The root mean square (RMS) of fluorescent intensity of TFF1, which indicates the intensity deviation from the mean value.

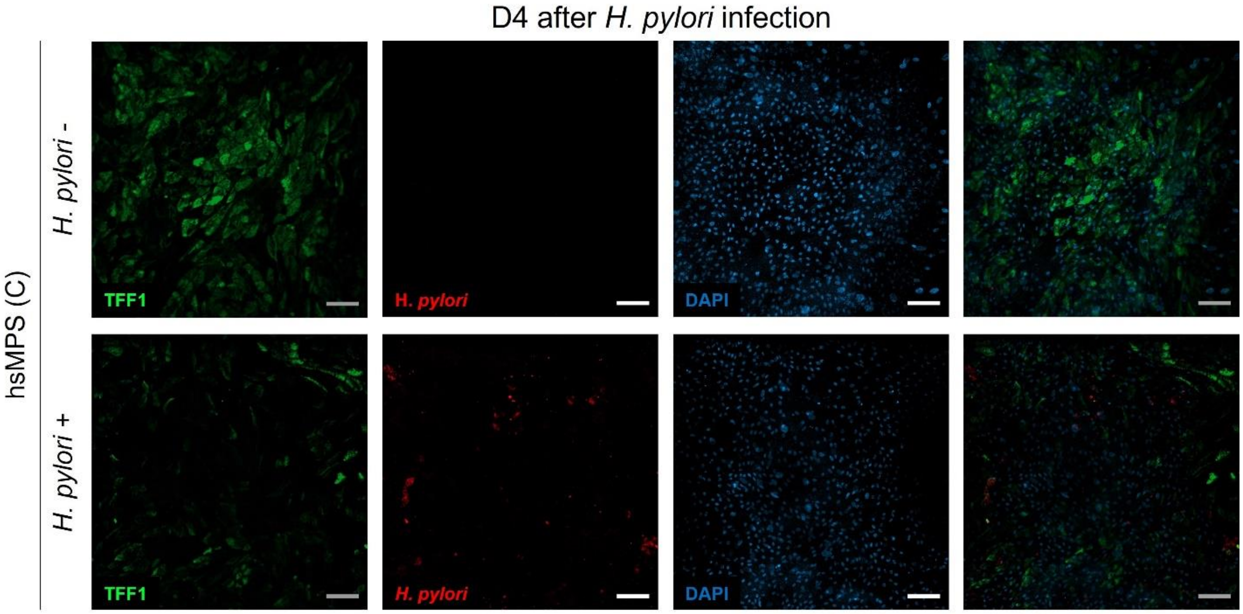

Supplementary Figure S7. Immunofluorescence micrographic images of the human gastric epithelium labeled with TFF1 (green) and *H. pylori* (red) of a noninfected (top) and a hsMPS infected with *H. pylori* for 4 days at an MOI of 10 (Scale bar, 100 μm).

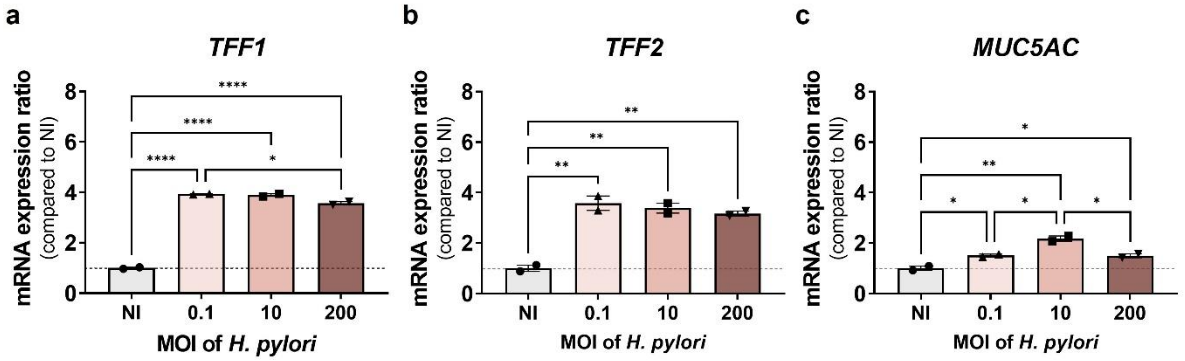

Supplementary Figure S8. The secretion of gastric protective peptides against *H. pylori* infection. The mRNA expression ratios of TFF1 (a), TFF2 (b), and MUC5AC (c) in gastric epithelial cells when infected with *H. pylori* at various MOI relative to non-infected control. The results are presented as the mean ± SEM. For statistical analysis, one-way ANOVA and Tukey's test was performed (**P* < 0.05; ***P* < 0.01; ****P* < 0.001; *****P* < 0.0001). MOI, Multiplicity of infection. NI, non-infected.

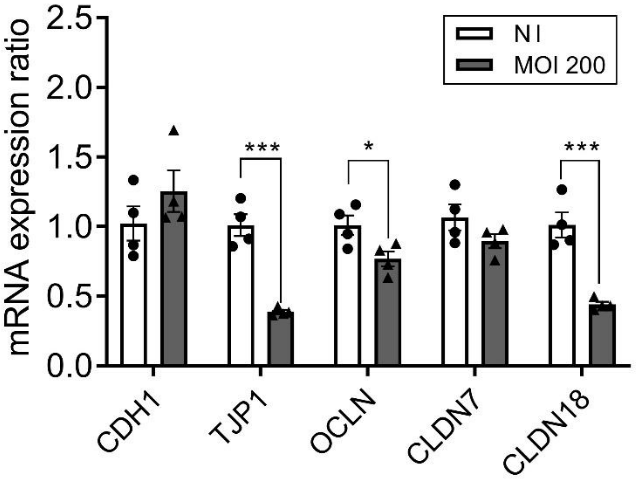

Supplementary Figure S9. The mRNA expression ratios of gene encoding tight junctional protein (E-cadherin, ZO-1, occludin, claudin-7, and claudin-18) in *H. pylori*-infected gastric epithelial cells at an MOI of 200 compared to non-infected (NI) control. The results are presented as the mean ± SEM. For statistical analysis, a Student's t-test was performed (*P < 0.05; **P < 0.01; ***P < 0.001; ****P < 0.0001). MOI, Multiplicity of infection.

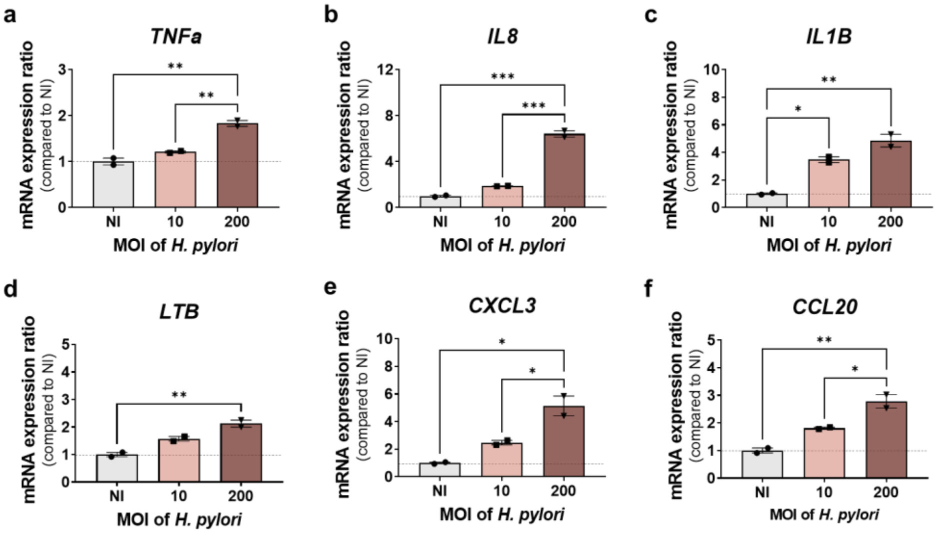

Supplementary Figure S10. *H. pylori*-induced inflammatory responses in hsMPS. The mRNA expression ratios of NF-κB-mediated inflammatory cytokines—TNFa (a), IL8 (b), IL1B (c), LTB (d), CXCL3 (e), CCL 20 (f) in gastric epithelial cells when infected with *H. pylori* at various MOI relative to noninfected control. The results are presented as the mean ± SEM. For statistical analysis, one-way ANOVA and Tukey's test was performed was performed. (*$P < 0.05$; **$P < 0.01$; ***$P < 0.001$; ****$P < 0.0001$). MOI, Multiplicity of infection. NI, non-infected. TNFa, tumor necrosis factor alpha; IL, interleukin; LTB, lymphotoxin beta; CCL/ CXCL, chemokine (C-C/C-X-C motif) ligand.

Table S1. Composition of expansion media (EM) and differentiation media (DM)

No.	Component	Manufacturer	Catalog no.	EM	DM
1	Advanced DMEM/F12	Gibco	12634028	+	+
2	L-WRN conditioned media (50%, v/v)	ATCC	CRL-3276	+	-
3	Noggin (100 ng/mL)	Peprotech	200-10C	-	+
4	Primocin (100 μg/mL)	InvivoGen	ant-pm-1	+	+
5	Plasmocin (5 μg/mL)	InvivoGen	ant-mpp	+	+
6	GlutaMAX (2 mM)	Gibco	35050061	+	+
7	HEPES (10 mM)	Welgene	15630106	+	+
8	Nicotinamide (10 mM)	Sigma	N3376	+	+
9	N-acetyl-L-cysteine (1.25 mM)	Sigma	A9165	+	+
10	B27 supplement (1X)	Gibco	17504044	+	+
11	Recombinant Human FGF10 (20 ng/mL)	Peprotech	100-26	+	+
12	Animal-Free Recombinant Human EGF (50 ng/mL)	Peprotech	AF-100-15	+	+
13	A83-01 (500 nM)	TOCRIS	2939	+	+
14	Gastrin - [Leu15]-Gastrin I human (10 nM)	Sigma	G9145	+	+
15	Y27632 (10 μM)	TOCRIS	1254	+	+
16	Fetal bovine serum (5%, v/v)	Merck	TMS-013-BKR	-	+

Table S2. List of ELISA kit

No.	Product name	Manufacturer	Catalog no.
1	Human protein Wnt-2b ELISA Kit	MyBioSource	MBS764038
2	Human EGF Quantkine ELISA Kit	R&D systems	DEG00
3	Human FGF-10 ELISA Kit	Cusabio	CSB-E14965h

Table S3. List of primer sequences used for qRT-PCR

**MUC5AC**	Forward	CAGCACAACCCCTGTTTCAAA	**NOTCH1**	Forward	TCAGCGGGATCCACTGTGAG
	Reverse	GCGCACAGAGGATGACAGT		Reverse	ACACAGGCAGGTGAACGACTTG
**MUC6**	Forward	CTGCCCTATACCAGCAATGGA	**NOTCH2**	Forward	ACAGTTGTGTCTGCTCACCAGGAT
	Reverse	CTGACCCATGTACTTCCGCTC		Reverse	GCGGAAACCATTCACACCGTTGAT
**TFF1**	Forward	CCCTCCCAGTGTGCAAATAAG	**NOTCH3**	Forward	GATGGCATGGATGTCAATGTTCGT
	Reverse	GAACGGTGTCGTCGAAACAG		Reverse	TGCCTCATCCTCTTCAGTTGGCAT
**TFF2**	Forward	CGGGGAGTGAGAAACCCTC	**NOTCH4**	Forward	TCAAACAGAGGTGGATGAGTGCCT
	Reverse	CACTGGAGTCGAAACAGCATC		Reverse	AGTTGGCCTTGTCTTTCTGGTCCT
**TNFα**	Forward	TCCCCAGGGACCTCTCTCTA	**DLL1**	Forward	TGTGACGAGTGTATCCGCTATCCA
	Reverse	GAGGGTTTGCTACAACATGGG		Reverse	AGGGCTTATGGTGTGTGCAGTAGT
**IL8**	Forward	ACACTGCGCCAACACAGAAAT	**DLL3**	Forward	TCCCGGATGCACTCAACAACCTAA
	Reverse	ATTGCATCTGGCAACCCTACA		Reverse	TTCAGGGCGATTCCAATCTACGGA
**IL1B**	Forward	AGCTACGAATCTCCGACCAC	**DLL4**	Forward	ACTGCGAGAAGAAAGTGGACAGGT
	Reverse	CGTTATCCCATGTGTCGAAGAA		Reverse	ACATGAGCCCATTCTCCAGGTCAT
**LTB**	Forward	GTACGGGCCTCTCTGGTACA	**JAG1**	Forward	ACTGCTCACCCTGAAAGACCACT
	Reverse	GTCCACCATATCGGGGTGAC		Reverse	AGGACCACAGACGTTCCAGGAAAT
**CXCL3**	Forward	CGCCCAAACCGAAGTCATAG	**JAG2**	Forward	TGCTGTGGAGGTGGCTATGTCT
	Reverse	GCTCCCCTTGTTCAGTATCTTTT		Reverse	TGTTTCCACCTTGACCTCGGT
**CCL20**	Forward	TGCTGTACCAAGAGTTTGCTC	**HES1**	Forward	GTCAACACGACACCGGATAAACCA
	Reverse	CGCACACAGACAACTTTTTCTTT		Reverse	TTTCCAGAATGTCCGCCTTCTCCA
**FOXL1**	Forward	GCCTCGCCCATGCTGTATC	**TJP1**	Forward	CAACATACAGTGACGCTTCACA
	Reverse	CGTTGAGCGTGACCCTCTG		Reverse	CACTATTGACGTTTCCCCACTC
**CDH1**	Forward	ATTTTTCCCTCGACACCCGAT	**CLDN7**	Forward	AGCTGCAAAATGTACGACTCG
	Reverse	TCCCAGGCGTAGACCAAGA		Reverse	GGAGACCACCATTAGGGCTC
**OCLN**	Forward	ACAAGCGGTTTTATCCAGAGTC	**CLDN18**	Forward	ACATGCTGGTGACTAACTTCTG
	Reverse	GTCATCCACAGGCGAAGTTAAT		Reverse	AAATGTGTACCTGGTCTGAACAG
**GAPDH**	Forward	TGTGGGCATCAATGGATTTGG			
	Reverse	ACACCATGTATTCCGGGTCAAT			

Table S4. List of antibodies used for immunofluorescence micrographic analysis and flow cytometric analysis

Primary antibody	Manufacturer	Catalog no.	Target name	Host
1	Invitrogen	MA5-12178	Mucin 5AC	Mouse
2	Santa cruz	sc-33668	Mucin 6	Mouse
3	Santa cruz	sc-28302	Gastrin	Mouse
4	Invitrogen	PA5-87974	LGR5	Rabbit
5	Proteintech	20874-1-AP	E-cadherin	Rabbit
6	Santa cruz	sc-23900	Ki67	Mouse
7	Proteintech	13734-1-AP	TFF1	Rabbit
8	Abcam	ab268118	TFF1	Mouse
9	Sigma	A22287	Phalloidin	-
10	Abcam	ab20459	*H. pylori*	Rabbit
11	Sigma	T7541	Acetylated tubulin	Mouse
12	Abcam	ab190226	FOXL1	Rabbit
13	R&D systems	FAB8078A	Human Lgr5/GPR49 APC-conjugated Antibody	Mouse
14	BosterBio	A01677-Dyl488	Anti-Human CCKBR DyLight® 488 Conjugated Antibody	Rabbit
15	MyBioSource	MBS4381712	anti-MUC5AC antibody conjugated to Biotin	Mouse
16	LSBio	LS-C723846	anti-MUC6 antibody conjugated to PE	Rabbit
17	Santa cruz	sc-17320	SOX2	Goat
**Secondary antibody**	**Manufacturer**	**Catalog no.**	**Product name**	**Host**
1	Invitrogen	A11001	Goat anti-Mouse IgG (H+L) Cross-Adsorbed Secondary Antibody, Alexa Fluor 488	Goat
2	Invitrogen	A110034	Goat anti-Rabbit IgG (H+L) Highly Cross-Adsorbed Secondary Antibody, Alexa Fluor 488	Goat
3	Invitrogen	A11012	Goat anti-Rabbit IgG (H+L) Cross-Adsorbed Secondary Antibody, Alexa Fluor 594	Goat
4	Abcam	ab150083	Goat Anti-Rabbit IgG H&L (Alexa Fluor® 647)	Goat
5	Invitrogen	S11223	Streptavidin, Alexa Fluor 488 conjugate	-
6	Invitrogen	A-11058	Donkey anti-Goat IgG (H+L) Cross-Adsorbed Secondary Antibody, Alexa Fluor 594	Donkey
